# Microwave-assisted synthesis of [^52^Mn]Mn-porphyrins: Applications in cell and liposome radiolabelling

**DOI:** 10.1016/j.nucmedbio.2022.08.006

**Published:** 2022

**Authors:** Peter J. Gawne, Sara M.A. Pinto, Karin M. Nielsen, George P. Keeling, Mariette M. Pereira, Rafael T. M. de Rosales

**Affiliations:** a School of Biomedical Engineering and Imaging Sciences, King's College London, St Thomas' Hospital, London, UK; b Department of Chemistry, University of Coimbra, 3004-535 Coimbra, Portugal

**Keywords:** Radiochemistry, Porphyrins, Cell labelling, Liposomes, PET, Manganese-52

## Abstract

**Background:**

Manganese porphyrins have several therapeutic/imaging applications, including their use as radioprotectants (in clinical trials) and as paramagnetic MRI contrast agents. The affinity of porphyrins for lipid bilayers also makes them candidates for cell/liposome labelling. We hypothesised that metalation with the positron emission tomography (PET) radionuclide ^52^Mn (*t*_1/2_ = 5.6 d) would allow long-term *in vivo* biodistribution studies of Mn-porphyrins, as well as a method to label and track cells/liposomes, but methods for fast and efficient radiolabelling are lacking.

**Results:**

Several porphyrins were produced and radiolabelled by addition to neutralised [^52^Mn]MnCl_2_ and heating using a microwave (MW) synthesiser, and compared with non-MW heating. MW radiosynthesis allowed >95 % radiochemical yields (RCY) in just 1 h. Conversely, non-MW heating at 70 °C for 1 h resulted in low RCY (0–25 % RCY) and most porphyrins did not reach radiolabelling completion after 24 h. Formation of the ^52^Mn-complexes were confirmed with radio-HPLC by comparison with their non-radioactive ^55^Mn counterparts. Following this, several [^52^Mn]Mn-porphyrins were used to radiolabel liposomes resulting in 75–86 % labelling efficiency (LE). Two lead [^52^Mn]Mn-porphyrins were taken forward to label MDA-MB-231 cancer cells *in vitro*, achieving *ca.* 11 % LE. After 24 h, 32–45 % of the [^52^Mn]Mn-porphyrins was retained in cells.

**Conclusions:**

In contrast to standard methods, MW heating allows the fast synthesis of [^52^Mn]Mn-porphyrins with >95 % radiochemical yields that avoid purification. [^52^Mn]Mn-porphyrins also show promising cell/liposome labelling properties. Our reported technique can potentially be exploited for the *in vivo* imaging of Mn-porphyrin therapeutics, as well as for the accurate *in vivo* quantification of Mn-porphyrin MRI agents.

## Graphical abstract

**Figure:**
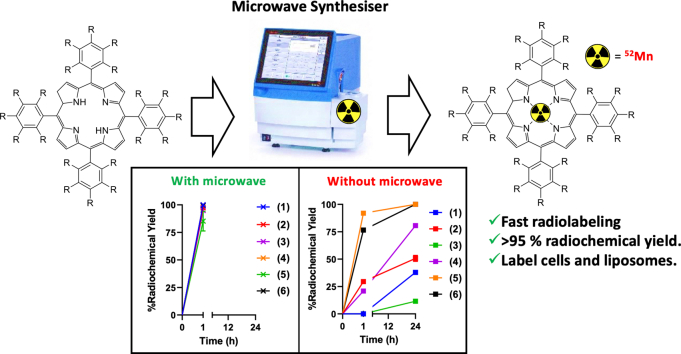

## 1 Introduction/background

**Fig. 1:**
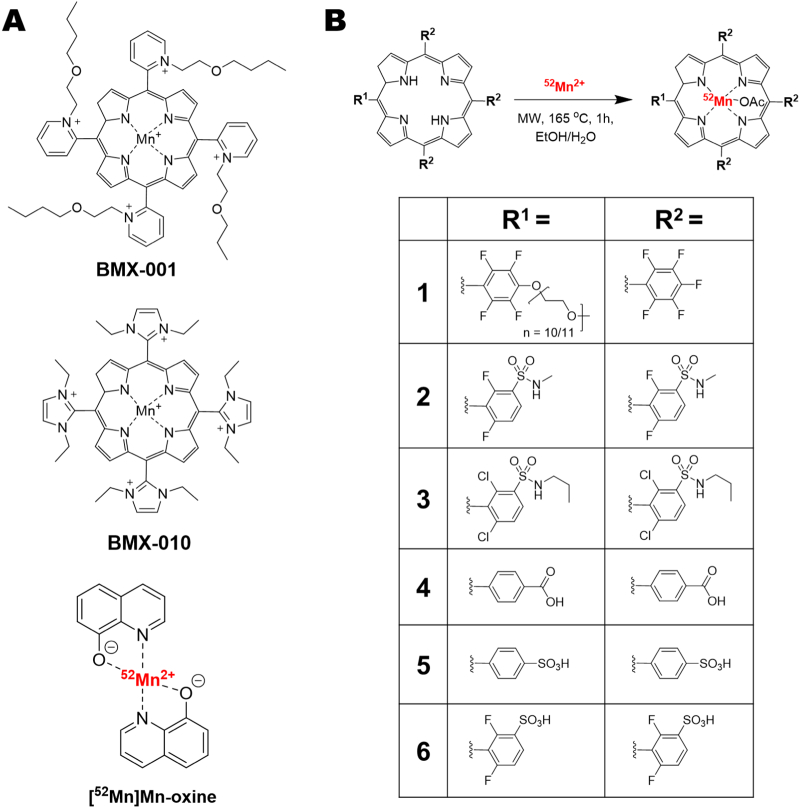
**A)** Chemical structures of the SOD mimetic Mn(III) porphyrins in clinical trials: BMX-001 (top) and BMX-010 (mid); and the cell/liposome radiolabelling agent [^52^Mn]Mn-oxine. **B)** Chemical structures of the porphyrins and their respective ^52^Mn complexes used in this work.

Porphyrins are a subtype of pyrrole-based macrocycles which – along with their metal complexes (metalloporphyrins) – have been studied for a wide variety of uses; including photochemistry and catalysis, and particularly as photosensitizers for photodynamic therapy applications [1], [2]. Whilst not naturally occurring in biology, manganese porphyrins have also been widely studied. When chelated, manganese in porphyrin complexes can exist in the +2, +3 and +4 oxidation states – with Mn(III) complexes being the most stable and common. The tunability of the porphyrin ring structure allows it to be easily functionalised to modify the redox properties of the Mn metal centre. A good example of this involves the vast body of research exploring Mn(III) porphyrins as superoxide dismutase (SOD) mimetics [3], [4]. Two Mn(III) porphyrin SOD mimetics have reached the clinic: BMX-001 (Fig. 1A, top) is currently in phase 1/2 trials for the protection of healthy tissue during radiotherapy in patients with glioma (NCT02655601), and anal cancer (NCT03386500) [5], [6], [7]. Additionally, BMX-010 (Fig. 1A, mid) was previously explored as an anti-oxidant to reduce cell death during islet isolation for islet transplantation in diabetes treatment (NCT02457858) [8]. Other Mn(III) porphyrins, such as Mn-TBAP (structure: Mn-(**4**) in Fig. 1B) have been shown to have a variety of therapeutic effects [9]. Mn-TBAP – whilst not a SOD-mimetic – has shown reactivity with reactive nitrogen species (RNS) and has displayed efficacy in models of spinal cord injury [9], and acute viral infection [10].

As well as the therapeutic properties of Mn(III) porphyrins, the high spin d^4^ complexes are paramagnetic – allowing T_1_-weighted contrast with magnetic resonance imaging (MRI). Hence, Mn(III) porphyrins have been explored as alternatives to gadolinium-based contrast agents for MRI imaging [11], which include: redox active [12], [13], enzyme-activated [14], and zinc-responsive probes [15], [16] as well as theranostic MRI-detectable photothermal agents in the form of Mn-porphysomes [17]. Furthermore, the lipid bilayer affinity and/or cell-permeability of Mn(III) porphyrins allows them to be used as cell and liposome labelling agents. Indeed several groups have designed MRI-based cell labelling and tracking agents using various Mn(III) porphyrins [18], [19]. However, despite the exceptional spatial resolution and versatility of MRI, *in vivo* cell/liposomal nanomedicine tracking using this imaging modality can be hindered by the limited field of view, challenging quantification and low sensitivity (in terms of concentration of administered contrast agent required for imaging). Hence, recent focus has shifted towards the development of positron emission tomography (PET) tracers for cell and liposome tracking; due to its high sensitivity and ability to produce longitudinal, quantifiable images on the whole body scale [20], [21], [22].

Our group has recently explored the relatively new manganese PET radioisotope [23], ^52^Mn (*t*_1/2_ = 5.59 days, β+ = 29.6 %) for radiolabelling of liposomes and cells using the compound [^52^Mn]Mn(oxinate)_2_ ([^52^Mn]Mn-oxine; Fig. 1A, bottom) [24], [25]. We hypothesised that the well-established chemistry, high stability, and membrane-permeability of Mn(III) porphyrins make them ideal candidates to be explored as cell and liposome labelling agents with ^52^Mn – as well as other biomedical applications. However, few examples of the synthesis of radiomanganese porphyrins exist in the literature [26], [27], with no studies using ^52^Mn. To address this, herein we describe a new method for the radiochemical synthesis of [^52^Mn]Mn-porphyrin complexes using six porphyrin ligands with various lipophilicities and charges (Fig. 1B), and then assessed their liposome labelling properties. Lead compounds were chosen and used for the labelling of cells, in comparison with our previously developed cell/liposome radiolabelling agent ([^52^Mn]Mn-oxine), to evaluate this approach.

## 2 Results

### 2.1 Synthesis of the porphyrin ligands and non-radioactive Mn-porphyrins

**Scheme 1:**
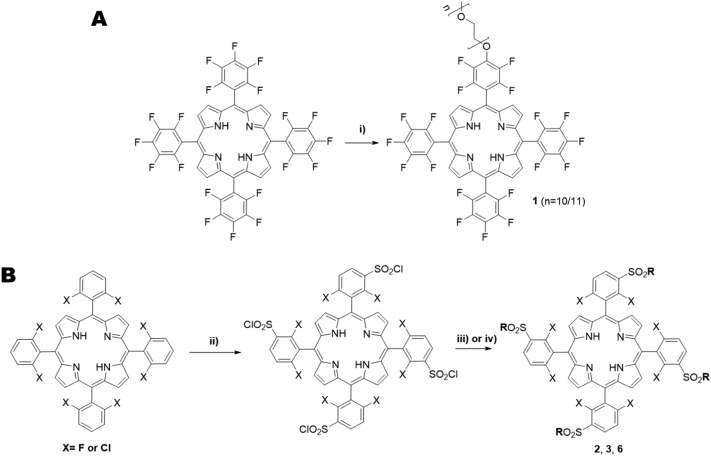
Reagents and reaction conditions: **A)** i) 48 h, 80 °C **B)***:* ii) if X = F: HSO_3_Cl, 2 h, 100 °C; if X = Cl: HSO_3_Cl, 3 h, 100 °C; iii) **2** (X = F), CH_3_NH_2_, CH_2_Cl_2_, RT, 3 h; **3** (X = Cl), CH_3_(CH_2_)_2_NH_2_, CH_2_Cl_2_, R.T., 3 h; iv) **6** (X = F), H_2_O, reflux, 12 h.

For the synthesis of the desired porphyrin ligands, a synthetic pathway involving their peripheral functionalization *via* nucleophilic substitution and complexation with manganese(II) acetate, was chosen. First, starting porphyrins were obtained *via* nitrobenzene methodology [28], where one-pot condensation of pyrrole with suitable benzaldehydes is achieved, using acetic acid/nitrobenzene as solvent and oxidant. After work-up, all starting porphyrins were obtained in yields similar to those found in the literature [29], [30]. Then, in the case of porphyrin **1,** nucleophilic substitution with the polyethylene glycol group was achieved by reacting the starting porphyrin with one equivalent of PEG500, DMF as solvent, and NaH as base, at 80 °C. The progress of the reaction was monitored *via* TLC and the reaction was stopped after 48 h. After purification using aluminium oxide column chromatography, porphyrin **1** was obtained in 48.5 % yield (Scheme 1A, further details in supporting information). Porphyrins **2** and **3** were prepared by previous chlorosulfonation of the corresponding porphyrin, followed by nucleophilic substitution with an amine derivative – in this case, methylamine and propylamine – giving **2** and **3** respectively, in similar yields to those found in literature [31]. Porphyrin **6** was prepared by chlorosulfonation followed by hydrolysis, providing **6** in 91 % yield (Scheme 1B). Porphyrins **4** & **5** were purchased from commercial sources.

Next, to obtain the non-radioactive Mn(III)-porphyrin complexes, porphyrins **1–3, 5** & **6** were reacted with manganese(II) acetate, and Mn-(**4**) was purchased from commercial sources. For porphyrins **1** & **2**, a buffer mixture of acetic acid/sodium acetate was used as solvent and for **3**, **5** and **6**, DMF was used. These high boiling point solvents allowed us to achieve complexation without the use of a microwave. The reaction progress was monitored by UV–vis spectroscopy, and – if needed – additional Mn^2+^ salt was added to allow the reaction to go to completion. All complexes were obtained in good yields similar to those found in literature for similar complexes [32], [33]. UV–vis spectrometry (Fig. S1) and HPLC analysis (Fig. S2) was performed on all porphyrins as well as their Mn(III) complexes – which were further characterised using mass spectrometry (Figs. S8–S12).

### 2.2 Radiosynthesis and characterisation of the [^52^Mn]Mn-porphyrins

Following the synthesis of the porphyrin ligands and their respective non-radioactive manganese complexes, various ^52^Mn radiolabelling conditions were initially tested using porphyrin **2** (structure in Fig. 1B). This porphyrin was chosen due to its favourable solubility in aqueous and organic solvents (see Supplementary Table 1). Reaction at room temperature by simple mixing of the reagents (0.2 mM porphyrin **2** and neutralised ^52^Mn in ethanol and acetate buffer respectively) did not result in any radiolabelling for up to 6 h. Relatively mild reaction conditions were then tried using 0.2 mM of porphyrin **2** and heating at 65–80 °C for up to 2 h. However, no difference between the reaction mixture and that of free ^52^Mn was observed by instant thin-layer chromatography (iTLC) using 0.1 M citrate, pH 5 (Table 1) or by Log P analysis. Small amounts of conversion (radio-chemical yield, RCY = 15.6 ± 2.3 %) to the radio-complex occurred when the mixture was heated at 70 °C for 48 h. Increasing the porphyrin concentration to 0.5 mM, improved the RCY with 52.8 ± 3.2 % after 48 h. However, whilst the half-life of ^52^Mn permits long reaction times, these conditions are not clinically-viable and were considered sub-optimal.

**Table 1:** Summary of iTLC/TLC methods used for the characterisation of the [^**52**^Mn]Mn-porphyrins compared with buffered ^**52**^Mn along with the Log P values for each.

Compound tested	iTLC/TLC mobile phase used			Log P value
	iTLC: ethyl acetate	iTLC: 0.1 M citrate, pH 5	TLC: 5 % w/v NH_4_OAc in 1:1 MeOH/H_2_O	
Buffered ^52^Mn	R_f_ = 0	R_f_ = 1	R_f_ = 0	−2.4 ± 0.7
^52^Mn-(**1**)	R_f_ = 1	R_f_ = 0	n/a	1.8 ± 0.0
^52^Mn-(**2**)	R_f_ = 0.9	R_f_ = 0	R_f_ = 0.7–0.8	1.8 ± 0.2
^52^Mn-(**3**)	R_f_ = 1	R_f_ = 0	n/a	1.2 ± 0.1
^52^Mn-(**4**)	R_f_ = 0	R_f_ = 1	R_f_ = 1	1.4 ± 0.1
^52^Mn-(**5**)	R_f_ = 0	R_f_ = 1	R_f_ = 1	−2.4 ± 0.1
^52^Mn-(**6**)	R_f_ = 0	R_f_ = 1	R_f_ = 1	−2.1 ± 0.1

**Fig. 2:**
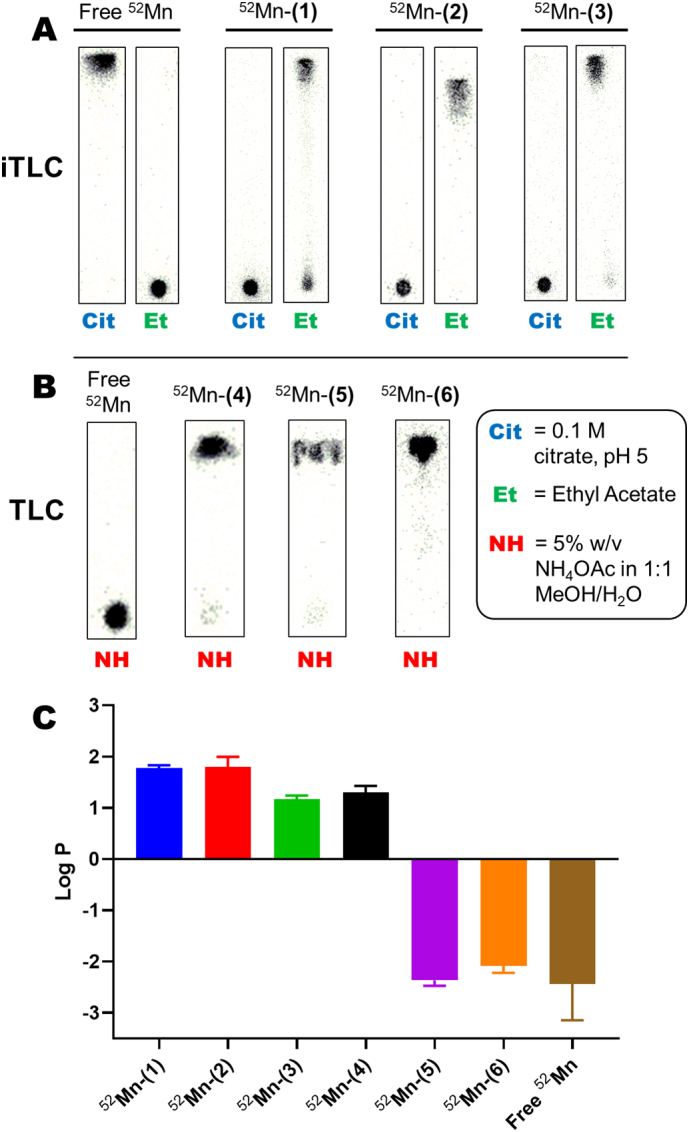
**iTLC/TLC and Log P characterisation of the [^52^Mn]Mn-porphyrin complexes**. A) iTLC characterisation for free ^52^Mn, ^52^Mn-(1), ^52^Mn-(2) and ^52^Mn-(3) using 0.1 M citrate (Cit) and ethyl acetate (Et) mobile phases. B) TLC characterisation of free ^52^Mn, ^52^Mn-(4), ^52^Mn-(5) and ^52^Mn-(6) using a 5 % w/v NH_4_OAc in 1:1 MeOH/H_2_O mobile phase. C) Log P values for the [^52^Mn]Mn-porphyrins and free ^52^Mn. Error bars represent mean ± SD, n = 3 for all compounds except for free ^52^Mn which is n = 2.

**Fig. 3:**
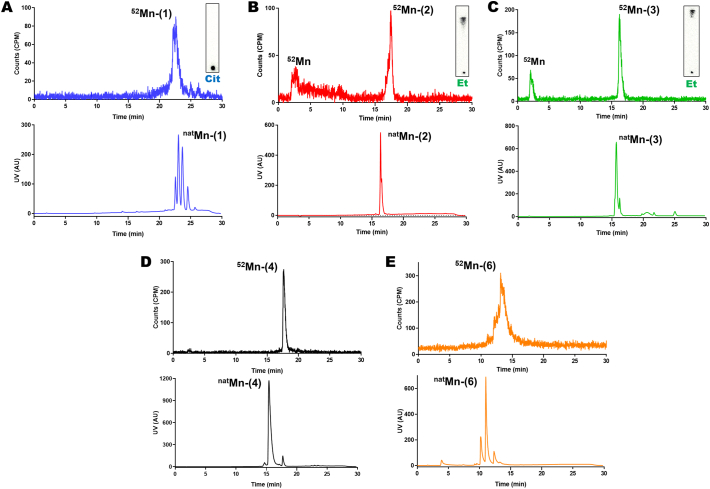
**Radio-HPLC of the [^52^Mn]Mn-porphyrin complexes synthesised using the microwave synthesis method**. Representative Radio-HPLC traces of A) ^52^Mn-(1); B) ^52^Mn-(2); C) ^52^Mn-(3); D) ^52^Mn-(4); E) ^52^Mn-(6); using the microwave synthesis method (top graph). The corresponding iTLC characterisation of the reaction of ^52^Mn-(1), ^52^Mn-(2) and ^52^Mn-(3) are also shown. In the case of ^52^Mn-(2) and ^52^Mn-(3) the presence of free ^52^Mn is clearly observed in both the iTLC and radio-HPLC, validating the two methods. HPLC traces of the corresponding non-radioactive Mn-porphyrins are pictured below the radio-HPLC.

Instead, we performed the same reaction using a microwave synthesis unit (MW; Biotage® Initiator+). A 90 % EtOH solvent system was chosen to avoid precipitation of the porphyrin ligand. This also allowed heating of the solution up to 165 °C in the MW synthesiser, which greatly improved the reaction times. After 1 h at 165 °C, the RCY for ^52^Mn-(**2**) was found to be 79.7 ± 1.8 %. iTLC conditions were found to move and to retain the suspected ^52^Mn complex on the baseline. Using an ethyl acetate mobile phase gives an Rf = 0.9 (Rf = 0 for free ^52^Mn) whereas by using 0.1 M citrate buffer the Rf = 0 (Rf = 1 for free ^52^Mn, Table 1, Fig. 2A). Log P analysis confirmed the presence of the lipophilic compound (log P for ^52^Mn-(**2**) = 1.8 ± 0.2) compared to that of just ammonium acetate (pH 7) buffered ^52^Mn (log P = −2.4 ± 0.7). Radio-HPLC analysis was carried out to confirm that the product was the ^52^Mn-(**2**) complex. Fig. 3B shows the radio-HPLC trace along with the UV trace for ^52^Mn-(**2**). The presence of *ca.* 20 % unreacted ^52^Mn could be seen eluting *ca.* 2 min, which matched the amounts observed by iTLC. Additionally, the peak in the radio-trace of ^52^Mn-(**2**) at 17 min 20 s closely followed that of non-radioactive Mn-(**2**) which elutes at 16 min 20 s, suggesting that the Mn(III) complex was being formed – taking into account the lag time between the two detectors. Finally, the MW synthesis conditions were further optimised by increasing the porphyrin concentration to 0.6 mM which resulted in RCY = 97.3 ± 0.9 % (iTLC trace in Fig. 2A).

The optimised MW synthesis method was then applied to other porphyrin ligands of varying lipophilicity and different functional groups (structures **1–6** in Fig. 1B), and initial characterisation carried out using iTLC and log P. After MW reaction for 1 h, iTLC analysis using ethyl acetate and citrate as a mobile phase showed the formation of a lipophilic compound with porphyrins **1** & **3** (Table 1, Fig. 2A). However, no change was observed with these methods for the remaining ligands, which behaved identically to ^52^Mn – both with iTLC and log P (Table 1). We hypothesised that the ^52^Mn-complexation was occurring, but was not detectable with these iTLC conditions. To elucidate this, we then performed characterisation using TLC plates with a 5 % w/v NH_4_OAc in 1:1 MeOH/H_2_O mobile phase. Using these conditions, the suspected ^52^Mn-(**4**) – ^52^Mn-(**6**) were shown to move with the solvent front, whilst unchelated ^52^Mn remains on the baseline (Table 1, Fig. 2B). The log P values of the ^52^Mn complexes were also calculated. The hydrophilicity of the sulfonated complexes, ^52^Mn-(**5**) and ^52^Mn-(**6**), was demonstrated with negative log P values similar to free ^52^Mn (−2.4 ± 0.1, −2.1 ± 0.1 and −2.4 ± 0.7 respectively; see Table 1 and Fig. 2C). The most lipophilic compounds were ^52^Mn-(**1**) and ^52^Mn-(**2**) (Log P = 1.8 ± 0.0 and 1.8 ± 0.2 respectively) followed by ^52^Mn-(**4**) and ^52^Mn-(**3**) with log P values of 1.4 ± 0.1 and 1.2 ± 0.1 respectively.

Radio-HPLC analysis was carried out on the remaining ^52^Mn-complexes; Fig. 3A & C shows the trace for ^52^Mn-(**1**) and ^52^Mn-(**3**) respectively. Elution times matched well for both of these complexes; again indicating that the reaction was forming the Mn(III) complexes of the respective ligands. As with ^52^Mn-(**2**), the presence of free ^52^Mn was observed with ^52^Mn-(**3**) which matched the iTLC results. Fig. 3D & E shows the radio-HPLC traces for ^52^Mn-(**4**) & ^52^Mn-(**6**) and Fig. S4 shows the radio-HPLC trace for ^52^Mn-(**5**). A larger difference (*ca.* 3 min) in elution between the non-radioactive and ^52^Mn-complexes was observed for ^52^Mn-(**4**) and ^52^Mn-(**6**). However, the presence of unchelated ^52^Mn was absent for these compounds.

**Fig. 4:**
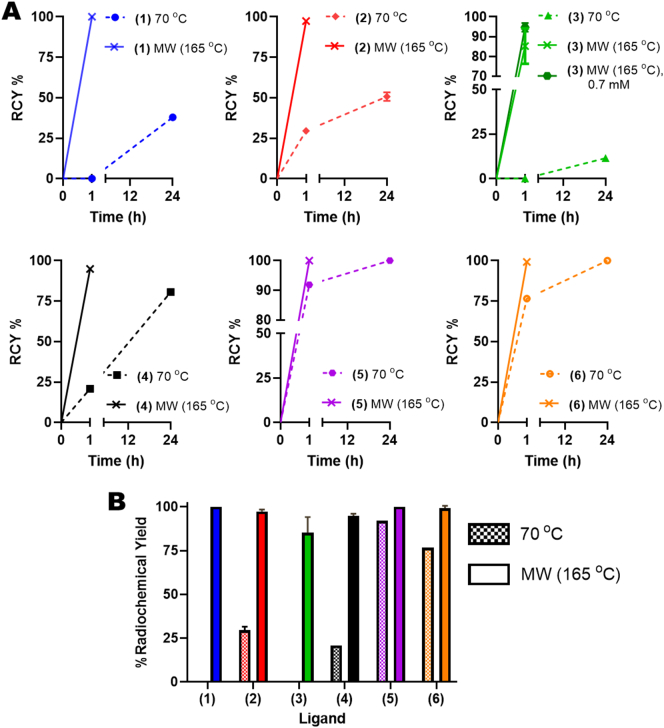
**Summary of the % radiochemical yields (RCY%) of the [^52^Mn]Mn-porphyrins**. A) Graphs showing a comparison of the radiochemical yields achieved overtime for the microwave synthesis method (solid lines) compared to that achieved with labelling at 70 °C (dotted lines) for porphyrins 1–6. For porphyrin 3 a further comparison is shown with the MW method at 0.7 mM compared to 0.6 mM used in all other experiments. B) Direct comparison between the radiochemical yields achieved for porphyrins 1–6 after 1 h heating at 70 °C (chequered bars) *versus* the microwave method (solid colour bars). Additionally, the graph also highlights the large differences in radiolabelling with porphyrins 1–6 after 1 h heating at 70 °C. Error bars represent mean ± SD.

Finally, to confirm the benefits of the MW labelling method compared with standard heating, the ^52^Mn complexations of all the porphyrins were carried out at 70 °C up to 24 h and the RCY % compared with the MW method at the same porphyrin concentration (Fig. 4A). After heating for 1 h, the RCY with the MW method was higher for every porphyrin ligand compared with heating at 70 °C (Fig. 4A & B). RCY % of >95 % could be achieved with the MW method, except for porphyrin **3**. However, by increasing the porphyrin concentration in the reaction mixture from 0.6 to 0.7 mM, the RCY increased from 85.2 ± 7.3 % to 94.7 ± 1.8 %. After 24 h at 70 °C, only complexation of porphyrins **5** and **6** had gone to completion, with the RCY after 1 h equal to 92 % and 77 %, respectively. One potential reason for these differences is the increased aqueous solubility of these porphyrins; allowing the reaction at 70 °C to be performed in H_2_O.

### 2.3 Liposome radiolabelling with the [^52^Mn]Mn-porphyrins

**Fig. 5:**
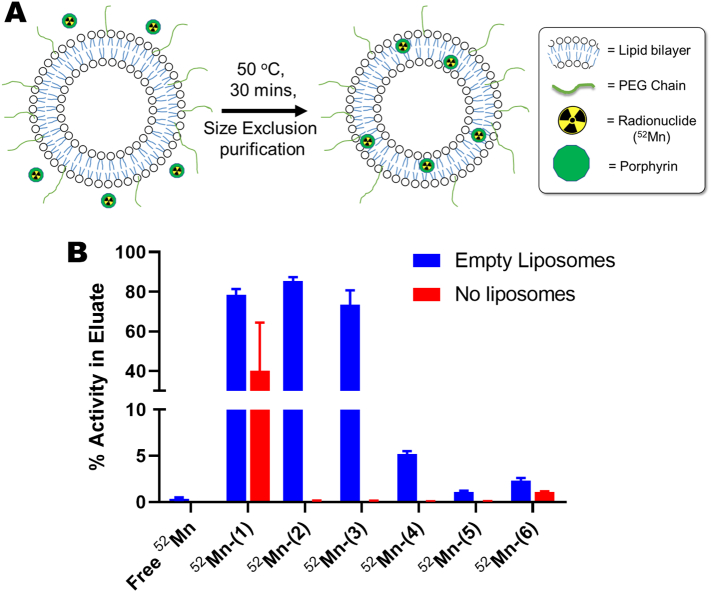
**Summary of the liposome labelling properties of the [^52^Mn]Mn-porphyrins**. A) Scheme showing the procedure, and potential mechanism, of liposome labelling using the [^52^Mn]Mn-porphyrins discussed in this work. B) Graph showing the liposome labelling properties of each of the ^52^Mn-labelled porphyrins compared with unchelated ^52^Mn. % Activity in the eluate represents the amount of activity that elutes with the liposomes (blue bars) or in the case of no liposomes (red bars) indicates the extent to which the [^52^Mn]Mn-porphyrins elute through the size exclusion columns. Error bars = mean ± SD n = 3. (For interpretation of the references to colour in this figure legend, the reader is referred to the web version of this article.)

After establishing a fast and reliable radiosynthetic method for the formation of the [^52^Mn]Mn-porphyrins, the liposome labelling properties of each of the complexes was tested. The labelling protocol was an adapted version of a previously reported method [24], [25], and was used with empty (no drug cargo) ‘DOXIL-like’ PEGylated liposomes (Fig. 5A). The [^52^Mn]Mn-porphyrins were incubated with the liposomes for 30 min at 50 °C and the mixture purified by size exclusion to separate the radiolabelled liposomes from the porphyrin complexes. This procedure was first tested without the presence of liposomes ([^52^Mn]Mn-porphyrins only), to demonstrate that the [^52^Mn]Mn-porphyrins do not elute from the size exclusion column in the liposome fraction. Only ^52^Mn-(**1**) showed activity (40.0 ± 24.4 %) in the eluent (Fig. 5B). Fig. 5B summarises the liposome radiolabelling properties of each of the [^52^Mn]Mn-porphyrins. The lipophilic compounds ^52^Mn-(**2**), ^52^Mn-(**1**) and ^52^Mn-(**3**) had the highest labelling efficiencies with 85.5 ± 1.8 %, 78.4 ± 3.0 % and 73.6 ± 7.1 %, respectively (Fig. 5B). Alternatively, as expected, the hydrophilic porphyrin complexes ^52^Mn-(**5**) and ^52^Mn-(**6**) showed the lowest liposome labelling (1.1 ± 0.1 % and 2.3 ± 0.3 % respectively (Fig. 5B). Interestingly, despite being highly lipophilic (Log P = 1.35), ^52^Mn-(**4**) showed relatively low loading into liposomes with 5.2 ± 0.3 % LE. The labelling efficiency of unchelated ‘free ^52^Mn’ was found to be 0.4 ± 0.1 % indicating that the porphyrin ligands facilitate the loading of the ^52^Mn into the liposomes.

### 2.4 Direct cell radiolabelling with the [^52^Mn]Mn-porphryins

Due to their favourable liposome labelling properties, ^52^Mn-(**1**) and ^52^Mn-(**2**) were taken forward for cell labelling. Despite its high liposome labelling, ^52^Mn-(**3**) was not taken further due to issues with precipitation of the porphyrin ligand observed during incubation with the liposomes; this is potentially exacerbated by the larger amounts of porphyrin **3** needed for the radiosynthesis of ^52^Mn-(**3**) (Fig. 4). The stability of ^52^Mn-(**1**) and ^52^Mn-(**2**) in serum-depleted cell medium was tested to ensure the complexes would remain intact in the cell radiolabelling conditions. The Log P of the [^52^Mn]Mn-porphyrins in the medium at room temperature was measured over time. These measurements showed that the lipophilic complexes were still present up to 24 h (Fig. S3A), which was considered adequate as the incubation time with cells is 30 min.

**Fig. 6:**
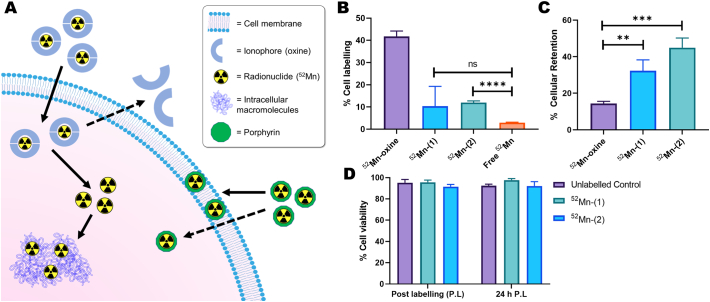
**Cell labelling properties of ^52^Mn-(1) and ^52^Mn-(2) compared with [^52^Mn]Mn-oxine**. A) Schematic summarising of the main methods for cell radiolabelling discussed in this work. An ionophore ligand forms a complex with a radionuclide (^52^Mn) which allows it to cross cell membranes. Once inside the cell, the radioisotope is released and trapped by binding to intracellular macromolecules. Alternatively, the surface of cells can be radiolabelled using stable radiopharmaceuticals which can interact with the lipid membrane (*e.g.* porphyrins). B) Graph showing the % cell labelling of ^52^Mn-(1) and ^52^Mn-(2) with MDA MB-231 cells compared with [^52^Mn]Mn-oxine and unchelated ^52^Mn. Unpaired *t*-test performed using GraphPad Prism. **** P ≤ 0.0001 C) Graph showing the cellular retention of ^52^Mn in MDA MB-231 cells labelled with [^52^Mn]Mn-oxine, ^52^Mn-(1) and ^52^Mn-(2) after 24 h. Unpaired t-test performed using GraphPad Prism. ** P = 0.0068 *** P = 0.0006 D) Graph showing the cell viability of MDA MB-231 cells labelled with ^52^Mn-(1) and ^52^Mn-(2) compared with unlabelled controls just after labelling and at 24 h. Error bars represent mean ± SD (n = 3).

The cell labelling properties of ^52^Mn-(**1**) and ^52^Mn-(**2**) were then tested using MDA-MB 231 breast cancer cells, and compared with [^52^Mn]Mn(oxinate)_2_ ([^52^Mn]Mn-oxine; structure in Fig. 1A, bottom) [25], as a positive radiolabelling control – and with ‘free ^52^Mn’ used as a negative control. [^52^Mn]Mn-oxine radiolabels cells *via* the passive accumulation of the complex into cells followed by release of ^52^Mn intracellularly which is trapped by binding to intracellular macromolecules (Fig. 6A). Alternatively, as with liposomes, we hypothesised that the [^52^Mn]Mn-porphyrins would intercalate in the lipid bilayer on the cell membrane (Fig. 6A). Hence, by testing the cell labelling of [^52^Mn]Mn-oxine and [^52^Mn]Mn-porphyrins, this also serves as a comparison between these two direct cell labelling methods. After 30 min incubation with the MDA-MB 231 cells, [^52^Mn]Mn-oxine showed the highest uptake into cells with a % labelling efficiency (LE) of 41.8 ± 2.0. The two [^52^Mn]Mn-porphyrins both showed lower labelling with 12.0 ± 0.6 % LE and 10.3 ± 7.3 % LE for ^52^Mn-(**2**) and ^52^Mn-(**1**) respectively (Fig. 6B). However, their % LE was still larger than that of unchelated ^52^Mn (2.9 ± 0.2 % LE), albeit only statistically significant in the case of ^52^Mn-(**2**) (P *≤* 0.0001). Additionally, small amounts of precipitation – presumably of the porphyrin ligand – were observed upon addition with both [^52^Mn]Mn-porphyrins. However this precipitate could be removed after radiolabelling during the cell washing step.

Despite their less favourable cell labelling properties compared to [^52^Mn]Mn-oxine, the cellular retention of ^52^Mn when using the [^52^Mn]Mn-porphyrins showed a significant improvement. Whereas for [^52^Mn]Mn-oxine only 14.4 ± 1.0 % of activity remained in cells after 24 h, this value was 32.3 ± 4.8 % and 44.9 ± 4.3 % for ^52^Mn-(**1**) and ^52^Mn-(**2**) respectively (Fig. 6C). At time points immediately after cell labelling and after 24 h post-labelling, no significant reduction in cell viability (based on trypan blue staining) was seen with the [^52^Mn]Mn-porphyrins compared with unlabelled controls (Fig. 6D).

## 3 Discussion

In this work, we have explored the radiochemical synthesis of manganese porphyrins using the PET radionuclide ^52^Mn (*t*_1/2_ = 5.59 days, β+ = 29.6 %). Whilst porphyrins have been radiolabelled with a wide variety of PET, SPECT and therapeutic radionuclides (*e.g.* ^68^Ga, ^64^Cu, ^111^In & ^177^Lu) [34], [35], to the best of our knowledge, only two studies exploring the synthesis of radiomanganese porphyrin complexes exist. Escanye *et al.* labelled a hematoporphyrin derivative with the non-imaging radionuclide, ^54^Mn (*t*_*1/2*_ = 313 d, 100 % γ) [26], and Klein *et al.* reported the ^51^Mn (*t*_*1/2*_ = 46 min, 97 % β^+^) complex of ligand **5** (Mn-TPPS_4_) [27]. However, these studies focused on single specific porphyrin ligands. Therefore, a versatile, procedure for the synthesis of radiomanganese porphyrins with a wide variety of chemical properties (*e.g.* charge, lipophilicity, varying functional groups) is still lacking. To address this need, we have developed a fast, robust method for the formation of radiomanganese porphyrins using a microwave synthesis unit.

Porphyrin **2** (Fig. 1B) was chosen as an ideal ligand for screening radiosynthesis conditions; due to its favourable solubility in both aqueous and organic solvents. Initial tests showed that under relatively mild conditions (70 °C, 0.2 mM ligand) no reaction occurred as observed by iTLC and log P measurements for up to 2 h. Incomplete conversion to ^52^Mn-(**2**) was achieved (RCY = *ca.* 16 %) by increasing the ligand concentration to 0.5 mM, but only after heating at 70 °C for 48 h. Hence, the use of a microwave synthesiser – which is a well-established method to aid the metalation of porphyrins [36], [37] – was employed to aid chelation. Using this method, >95 % RCY could be achieved after 1 h at 165 °C and by increasing the porphyrin concentration to 0.6 mM. These high RCYs were also achieved when applied to the other porphyrin ligands, except for porphyrin **3** – which required a concentration of 0.7 mM to attain the same RCY. A comparison to heating at 70 °C, showed that the microwave synthesis method was superior for every porphyrin ligand tested (Fig. 4). Only the complexation of porphyrins **5** and **6** went to completion after 24 h. One potential reason for these differences is the increased aqueous solubility of these porphyrins; allowing the reaction at 70 °C to be performed in H_2_O – as opposed to buffer/EtOH. The higher boiling point of this solvent may have resulted in more preferable complexation reaction conditions. An increase in reaction kinetics by increasing temperature was also observed by Klein *et al.* [27]. Quantitative radiolabelling of [^51^Mn]Mn-TPPS_4_ (identical chemical structure to Mn-(**5**)) could be achieved after 2 min after heating at 108 °C and after 10 min at 85 °C. After 15 min heating at 65 °C and 45 °C the RCY was *ca.* 70 % and *ca.* 10 %, respectively. By comparison, ^52^Mn-(**5**) was prepared with a RCY of 92 % after 1 h at 70 °C, and with complete conversion at 165 °C using the MW synthesis method. Whilst our labelling procedure is slightly longer than that described by Klein *et al.*, the concentration of porphyrin used in the reaction is almost a magnitude lower than that reported for the synthesis of [^51^Mn]Mn-TPPS_4_ (0.6 mM *vs.* 5 mM respectively) [27]. Additionally, our results show that the MW method is able to radiolabel a wide variety of porphyrins with varying functional groups, overall charge and lipophilicities.

Along with iTLC and Log P characterisation of our MW synthesis of the [^52^Mn]Mn-porphyrins, radio-HPLC characterisation was also performed with the ^52^Mn-complexes – with a comparison to the synthesised non-radioactive ^nat^Mn-porphyrin counterparts (Fig. 3). Elution times of ^52^Mn-(**1**) – ^52^Mn-(**3**) (Fig. 3A–C) matched well with their non-radioactive metal complexes – taking into account the lag time between the UV and radio detectors – suggesting that the reaction was forming the Mn(III) complexes of the respective ligands. In the case of ^52^Mn-(**4**) – ^52^Mn-(**6**), the radio-HPLC traces showed a larger difference (*ca.* 3 min) in elution between the non-radioactive and ^52^Mn-complexes. However, the presence of unchelated ^52^Mn was absent for these compounds. It is possible that the differences seen for these compounds are due to the formation of the uncharged, less hydrophilic Mn(II) porphyrin complex occurring in the radiochemical reaction. Additionally, tetra-arylporphyrins with substituents in the ortho- position – such as ligands **2, 3** and **6** can have several atropisomers [38], [39]. Also, in the case of ligand **1**, a polydisperse mixture is present that can affect the HPLC signal. Hence, this may explain the broad peaks observed on the radio-HPLC traces with ^52^Mn-(**1**) and ^52^Mn-(**6**) – as well as differences in retention time for the latter compound. Furthermore, these likely are the source of the multiple peaks observed on the HPLC traces for several of the ligands and non-radioactive complexes (Fig. S2).

Whilst we did not comprehensively study the stability of the [^52^Mn]Mn-porphyrins used in this work, Log P analysis indicated that ^52^Mn-(**1**) and ^52^Mn-(**2**) were stable in aqueous buffer over 24 h (Fig. S3A). Additionally, ^52^Mn-(**1**) also appeared to be unstable in human serum with *ca.* 75 % of the radioactivity associated with serum after 24 h (Fig. S3B). However, the ethanol precipitation method used cannot distinguish between ^52^Mn that has been trans-chelated by serum proteins, and intact ^52^Mn-(**1**) that is interacting with the serum. Both porphyrins and their Mn(III) complexes have been reported to bind with serum proteins, in particular albumin [40], [41], [42]. Furthermore, several groups have reported high serum stability of Mn-porphyrins. Whilst there is no reported data on the stability of Mn-(**1**), work carried out using the Mn-TPPS/Mn-(**5**) showed that the complex was stable in human plasma for up to 9 days based on UV-spectroscopy [43]. Bohdiewicz et al. also looked at the *in vivo* stability of a Mn-hematoporphyrin complex. UV-spectroscopy analysis of urine and serum of rats 24 h post-administration showed only the Mn-complex, with no presence of the free porphyrin observed [44]. However, the use of these techniques for determining stability is limited, as they cannot detect or quantify the amount of Mn present. In fact, the labelling method described above may also be used as a tool for assessing the stability of clinically relevant Mn-porphyrins in biological medium and *in vivo*.

Based on these results we propose that our MW radiosynthesis method would be highly valuable for the biological validation of Mn(III) porphyrin complexes. As with the methodology using organic radionuclides (*e.g.* ^11^C, ^18^F), our technique allows the synthesis of chemically identical radio-tracers (isotopologue) to assess the *in vivo* biodistribution of Mn(III) porphyrins and their bioconjugates. Several cationic Mn(III) porphyrins have been reported as SOD mimetics [4]. One agent BMX-001 (Fig. 1A) is currently in phase I/II clinical trials for use as a radioprotection agent in cancer treatments. Furthermore, we also prepared a radioactive analogue of Mn-TBAP (^52^Mn-(**4**)) which has also shown therapeutic effects in various disease models [9]. Our technique would allow a more detailed study of the biodistribution and pharmacokinetics for both of these agents. Finally, our technique could also be applied to the development of manganese-based PET/MRI tracers. Whilst the concentrations of manganese present in our [^52^Mn]Mn-porphyrin probes are inherently too low to allow contrast-imaging with MRI, our method may allow the *in vivo* validation of Mn-porphyrin based MRI tracers. Zhou *et al.* recently used ^52^Mn-labelled chelators to investigate the biodistribution and elimination of ^nat^Mn-based MRI agents [45]. Additionally, Vanasschen *et al.* proposed the radiolabelling of CDTA with isotopic mixtures of ^52^Mn/^nat^Mn to produce a bimodal imaging probe made up of two chemically identical contrast agents – one containing ^52^Mn for PET imaging, and the other containing ^nat^Mn for MRI contrast [46]. These approaches could be applied to Mn(III) porphyrin MRI agents to allow the assessment of the concentration of the probe based on PET imaging and *ex vivo* biodistribution [13].

Following the radiosynthesis of the [^52^Mn]Mn-porphyrins, their liposome labelling ability was tested using empty DOXIL-like liposomes. Liposomes containing Mn-porphyrins have been previously reported as potential contrast agents for MRI [17], [47]. Additionally, Luo *et al.* described a method of radiolabelling porphyrin-containing liposomes with ^64^Cu to allow tracking with PET [48]. However, each of these studies used porphyrin-phospholipid conjugates to form the liposomal particles. Hence cannot be directly compared to this work. To the best of our knowledge, Mn-porphyrins have yet to be used as ‘direct labelling’ agents for the imaging of liposomes; wherein liposomes are labelled without modification using stable complexes. As shown in Fig. 5A, we believe the lipophilic complexes are inserted into the lipid bilayer surface. Whilst, we have not demonstrated this directly in this work, several examples in the literature support this hypothesis. Firstly, the incorporation of lipophilic Mn-porphyrins into the surface of liposomes during their formation has been previously reported to occur [49], [50]. Additionally, previous studies using electron paramagnetic resonance spectroscopy have shown that Mn-porphyrins can indeed integrate into the lipid bilayer of liposomes [51], [52]. Of all the [^52^Mn]Mn-porphyrins tested, only the lipophilic ^52^Mn-(**1**) – ^52^Mn-(**3**) showed incorporation into the liposomes (Fig. 5B). The absence of labelling seen with the hydrophilic ^52^Mn-(**5**) and ^52^Mn-(**6**) complexes – which have a log P value of −2.4 and −2.1 respectively – suggests hydrophobicity of the complexes is necessary for labelling. However, in the case of ^52^Mn-(**4**) (log P = 1.4 ± 0.1), low levels of incorporation (*ca.* 5 %) were seen. It is likely that the buffered solution used during labelling caused deprotonation of the four carboxylate groups [53], forming a more hydrophilic complex with an overall −3 charge. The labelling procedure was also repeated in the absence of liposomes. Only ^52^Mn-(**1**) showed activity (40.0 ± 24.4 %) in the eluent (Fig. 5B) – despite the radio-HPLC only showing one lipophilic species (Fig. 3A). This size-exclusion chromatography analysis strongly suggests that ^52^Mn-(**1**) – at least partly – forms a large molecular weight species. The amphiphilic complex (with its long PEG chain) may allow the formation of micelles. However, the identity of this species is unknown with other possible candidates including aggregates or colloids. Future work will be carried out to investigate this interesting property of ^52^Mn-(**1**) further. Finally, we also demonstrated that unchelated ^52^Mn was unable to label the empty liposomes, indicating that the liposome incorporation of ^52^Mn is mediated by the porphyrin complex.

Due to their liposome labelling properties, the cellular labelling of ^52^Mn-(**1**) and ^52^Mn-(**2**) was tested in the MDA-MB-231 cell line, which was chosen as it was previously labelled with [^52^Mn]Mn(oxinate)_2_ ([^52^Mn]Mn-oxine; structure in Fig. 6A) [25], allowing its use as a positive radiolabelling control – with ‘free ^52^Mn’ used as a negative control. The [^52^Mn]Mn-porphyrins both had lower uptake than [^52^Mn]Mn-oxine (*ca.* 11 % for the [^52^Mn]Mn-porphyrins and *ca.* 42 % for [^52^Mn]Mn-oxine) after 30 min incubation, whilst showing increased uptake over unchelated ^52^Mn (*ca.* 3 %). This indicates that the porphyrin complexes are increasing the cell uptake of ^52^Mn, and that the cell labelling observed is not simply due to release of ^52^Mn from the porphyrin ring – followed by uptake of unchelated Mn. This matches the observation that the [^52^Mn]Mn-porphyrin complexes were stable in cell medium for up to 24 h (Fig. S3A). The incubation times were not optimised for the [^52^Mn]Mn-porphyrins and were instead chosen to match that of previous work with [^52^Mn]Mn-oxine [25]. Thus, a possibility is that cellular uptake of the ^52^Mn-complexes may be improved with longer incubation times. Dabrowski *et al.* demonstrated that the Zn complex of porphyrin **2** (Zn-(**2**)) showed time-dependent uptake in A549 cells with the maximum uptake occurring after 6 h incubation [54]. Additionally, Venter *et al.* labelled human embryonic stem cells with a Mn-porphyrin and found that a 2 h incubation was insufficient for labelling based on the difference in T1 relaxation times. This was improved by using a 24 h incubation time [18]. Whilst the physical long half-life of ^52^Mn makes increased incubation times feasible, this approach is not ideal when taken from a clinical *in vivo* cell tracking perspective. In this context, efficient cell labelling should be achieved as quickly as possible. Whilst the sub-cellular location of the [^52^Mn]Mn-porphyrins in this study was not established, integration in the cellular membrane may occur – as with liposomal membranes. Dabrowski and collaborators previously investigated the sub-cellular distribution of Zn-(**2**); showing it was partly membrane-bound, as well as being distributed throughout the cell [54]. However, a direct comparison with this work cannot be made due to the difference in cell lines and also due to the use of the Zn^2+^ complex, which will have an overall neutral charge. Further work will need to be carried out to determine the intracellular distribution of these compounds.

Despite their reduced uptake compared to [^52^Mn]Mn-oxine, the cellular retention of the [^52^Mn]Mn-porphyrins after 24 h was significantly higher. The low cellular retention of ^52^Mn when using oxine (*ca.* 14 %) was previously reported and is likely due to bioavailability of Mn causing trafficking of the metal out of the cell [25]. Conversely, after 24 h, 32 % of ^52^Mn-(**1**) and 45 % of ^52^Mn-(**2**) remained cell-bound. These results indicate that the [^52^Mn]Mn-porphyrins are at least partly retained by a different mechanism to that of [^52^Mn]Mn-oxine. However, partial release of ^52^Mn from the porphyrin ring cannot be ruled at this stage. Trypan blue staining indicated that no major cell death occurred both after radiolabelling and after 24 h with either of the [^52^Mn]Mn-porphyrins. Despite this, the cellular retention of the [^52^Mn]Mn-porphyrins tested does not justify the use of the long-lived ^52^Mn. Hence, we did not proceed further with cellular retention measurements over longer periods or with the radiolabelling of other, more clinically relevant, cell types – such as T-cells. Ideally, cellular retention should be higher and more sustained. For example, [^89^Zr]Zr-oxine has shown retention *ca.* >65 % in various cell lines after 24 h – with at least 30 % retention observed at later timepoints (5–7 days) [55], [56], [57].

## 4 Conclusions

In this work, a versatile and efficient method for the synthesis of [^52^Mn]Mn-porphyrins has been developed for the first time. By using a microwave synthesis unit, radiochemical yields >95 % can be achieved after just 1 h. The formation of the [^52^Mn]Mn-porphyrin can be shown using radio-HPLC or iTLC/TLC methods with a clear distinction between the radiocomplex and unchelated ^52^Mn. A screen of the [^52^Mn]Mn-porphyrins showed that lipophilic compounds ^52^Mn-(**1**), ^52^Mn-(**2**) and ^52^Mn-(**3**) were all capable of labelling empty DOXIL-like liposomes with labelling efficiencies >70 %. Cell labelling experiments with ^52^Mn-(**1**) and ^52^Mn-(**2**) showed that, whilst cell labelling efficiency with each of these compounds was lower than with [^52^Mn]Mn-oxine, the cellular retention was higher after 24 h. Based on these results, we believe that the methodology of using lipophilic [^52^Mn]Mn-porphyrins as cell labelling agents shows promise. However, new compounds need to be explored allowing higher incorporation and retention within cells. To the best of our knowledge, this is first time radio-porphyrin complexes have been explored as direct cell and liposome labelling agents. Future work will focus on the radiolabelling, *in vitro* serum stability and *in vivo* imaging of Mn(III) porphyrins used as therapeutic/MRI contrast agents. Additionally, new porphyrin structures more suitable for cell/liposome labelling will be investigated and their *in vivo* cell/liposome tracking ability tested using PET imaging.

## 5 Materials and methods

Chemicals: The following chemicals were purchased from Sigma Aldrich: dimethyl sulfoxide (DMSO), Human serum, 1-octanol, ammonium acetate (NH_4_OAc), acetic acid, manganese(II) acetate (Mn(OAc)_2_·4H_2_O), manganese(II) chloride (MnCl_2_·4H_2_O), 4,4′,4″,4‴-(Porphine-5,10,15,20-tetrayl)tetrakis(benzoic acid) (porphyrin **4**), Mn(*III*)tetrakis(4-benzoic acid)porphyrin chloride (Mn-(**4**)). Porphyrin **5** was purchased from Porphychem. Porphyrin **1**, **2**, **3** and **6** were obtained using previously described methodologies and characterisation data in good agreement [28], [29], [30], [31]. The following chemicals were purchased from Fisher Scientific: trifluoroacetic acid (TFA), acetonitrile (AcN), chloroform (CHCl_3_), ethyl acetate (EtOAc), ethanol (EtOH), dimethylformamide (DMF). PEGylated HSPC/CHOL Liposomes (empty DOXIL-like liposomes) were purchased from FormuMax Scientific, Inc. Water (18.2 MΩ·cm) was obtained from an ELGA Purelab Option-Qsystem. Phosphate buffered saline (PBS) tablets were purchased from Gibco.

Cell Culture: Dulbecco's Modified Eagle Medium (DMEM), Roswell Park Memorial Institute (RPMI) 1640 Medium, Fetal Bovine Serum (FBS), l-glutamine, Trypsin, Trypan Blue, Penicillin-Streptomycin were all purchased from Sigma Life Sciences. All cell culture flasks and well plates were purchased from Techno Plastic Products (TPP®). MDA-MB 231 cells were kindly provided by Dr. Marlies Glatz from the Department of Imaging Chemistry and Biology, King's College London.

Equipment: The UV titrations were carried out using a PerkinElmer Lambda 25 spectrometer, All other UV–vis measurements were carried out using a Thermo Scientific NanoDrop 2000c spectrophotometer with samples in Brand 70 μL micro cuvettes. Radioactivity in samples was measured using CRC-25R dose calibrator (Capintec). iTLC-SG and SA strips were purchased from Agilent, UK and scanned using the PerkinElmer Cyclone Plus Storage Phosphor Imager. Gamma counting was performed using a Wallac 1282 CompuGamma γ counter. SFCA Syringe filter, 0.2 μm, was purchased from Corning®. PD Mini-Trap G-25 size exclusion columns were purchased from GE Healthcare. Dynamic light scattering (DLS) and zeta-potential measurements were performed with a Zetasizer Nano ZS instrument (Malvern Instruments, UK) pH 0–14 indicator strips were purchased from Whatman™ Microwave synthesis was carried out using a Biotage® Initiator+ microwave synthesiser using Biotage® Microwave Reaction Vials (0.2–0.5 mL) Analytical reversed-phase HPLC was performed on an Agilent 1200 LC system using an Agilent Eclipse XDB-C18 column (4.6 × 150 mm, 5 μm) with UV detection at 254 nm, coupled to a LabLogic Flow-Count radioactivity detector with a sodium iodide probe (B-FC-3200).

### 5.1 Production of [^52^Mn]MnCl_2_

^52^Mn was prepared at the Hevesy Laboratory, Technical University of Denmark according to the procedure described by Fonslet et al. [58]. In brief, the manganese-52 was produced on a GE PETtrace cyclotron by 16 MeV proton irradiation (240 min, 20 μA) of a pressed natural chromium target. Separation of the ^52^Mn from the chromium target material was performed by four sequential anion exchange purifications, trapping the ^52^Mn out of ethanol–HCl mixtures and yielding the final [^52^Mn]MnCl_2_ product (approx. 350 MBq) in 0.1 M HCl.

### 5.2 Radiosynthesis of [^52^Mn]Mn-porphyrins

2 mg/mL stock solutions of all the [^52^Mn]Mn-porphyrins were prepared. Porphyrins **1, 2, 3** & **4** were dissolved in EtOH, whereas porphyrins **5** & **6** were dissolved in purified H_2_O.

#### 5.2.1 General method 1 (porphyrin **2** only) (standard heating)

An aliquot of [^52^Mn]MnCl_2_ (0.1–0.2 MBq) was neutralised with addition of 0.5 M NH_4_OAc solution. An aliquot of an ethanolic solution of porphyrin **2** (2 mg/mL) was then added and the solution made up to 100 μL of EtOH to give either a 0.2 or 0.5 mM porphyrin reaction concentration in 90 % EtOH. The reaction was then tested at varying ligand concentrations, temperatures and times (see Table S1).

#### 5.2.2 General method 2 (standard heating)

An aliquot of [^52^Mn]MnCl_2_ (0.1–0.2 MBq) was neutralised with addition of 0.5 M NH_4_OAc solution. An aliquot of an ethanolic solution of each porphyrin (2 mg/mL) was then added and the solution made up to 100 μL – EtOH (for porphyrins **1, 2, 3** & **4**) or H_2_O (for porphyrins **5** & **6**) – to give either a 0.6 mM porphyrin reaction concentration in 90 % EtOH (for porphyrins **1, 2, 3** & **4**) or H_2_O (for porphyrins **5** & **6**). The solution was then heated at 70 °C for 24 h.

#### 5.2.3 General method 3 (microwave reactor)

An aliquot of [^52^Mn]MnCl_2_ (0.1–3 MBq) was neutralised with addition of 0.5 M NH_4_OAc solution. An aliquot of an ethanolic/aqueous solution of the porphyrin ligands (2 mg/mL) was then added to a 0.5 mL microwave reaction vial containing a magnetic stirrer bar. The solution was then made up to 500 μL with EtOH or H_2_O – to give a 0.6 mM or 0.7 mM (porphyrin **3** only) porphyrin concentration in 90 % EtOH (for porphyrins **1, 2, 3** & **4**) or H_2_O (for porphyrins **5** & **6**). The reaction vial was sealed and placed in a Biotage® microwave synthesiser unit, and the solution was then heated at 165 °C for 1 h.

### 5.3 Characterisation of the [^52^Mn]Mn-porphyrins

iTLC: An aliquot (2 μL) of the [^52^Mn]Mn-porphyrin reaction solution was dotted onto an iTLC strip (silica gel, 10 cm length) and ran the appropriate mobile phase. The iTLC strip was then analysed using a phosphor imager with film.

TLC: As above but using a 6 cm length plate with a run length of 4 cm.

For a summary of the various iTLC and TLC conditions for each ligand see Table 1.

Log P: Purified H_2_O (25 mL) and 1-octanol (25 mL) were added to a falcon tube and shaken vigorously and the layers left to saturate for at least 24 h. An aliquot (500 μL) of each layer was then added to an Eppendorf. An aliquot (20 μL) of the [^52^Mn]Mn-porphyrin reaction mixture was then added to the two layers and vortexed for 5 min. After mixing, the suspension was vortexed briefly for 10 s to settle the layers, and 200 μL of each layer was extracted. The octanol and H_2_O layers were then gamma counted (Wallac CompuGamma) and the Log P was calculated as below:

$$\mathrm{Log}\;P=\mathrm{Log}\;\left(\frac{\text{Activity}\;\left(\mathrm{CPM}\right)\text{in octanol layer}}{\text{Activity}\;\left(\mathrm{CPM}\right)\text{in}\;\mathrm{water}\;\text{layer}}\right)$$

Reversed phase-HPLC: Approximately 0.1 MBq (25 μL) of the [^52^Mn]Mn-porphyrin solution was injected into the RP-HPLC system. For all Mn-porphyrins, solvent system 1 was used, except for Mn-**(3)** for which solvent system 2 was used. Column used: Eclipse XDB-C18 (5 μm; 4.6 × 150 mm). Flow rate: 1 mL/min.

### 5.4 Liposome labelling with the [^52^Mn]Mn-porphyrins

An aliquot of empty DOXEBO liposomes (63–150 μL, 2.7 mg, 2.66 μmol total lipid) was made up to 150 μL (if necessary) with saline. A 10 μL (20 kBq) aliquot of the appropriate [^52^Mn]Mn-porphyrin in DMSO (6.3 % v/v maximum) or H_2_O (^52^Mn-(**5**) and ^52^Mn-(**6**) only) was added to the liposomes and incubated at 50 °C for 30 min with occasional mild agitation of the solution. The reaction mixture (total volume 160 μL) was then added to a G-25 minitrap SE column, followed by saline (340 μL). The liposomes were then eluted with 750 μL of saline.

**For the [^52^Mn]MnCl_2_ control**: a 10 μL aliquot of [^52^Mn]MnCl_2_ buffered with 0.5 M NH_4_OAc was incubate with 150 μL DOXEBO.

**For the [**^**52**^**Mn]Mn-porphyrin only (No liposome) control:** A 10 μL (20 kBq) aliquot of [^52^Mn]Mn-porphyrin in DMSO or H_2_O (^52^Mn-(**5**) and ^52^Mn-(**6**) only) was incubated with a solution of saline (150 μL). Both controls then treated as above.

### 5.5 Cell labelling of MDA MB-231 cells using [^52^Mn]Mn-porphyrins and [^52^Mn]Mn-oxine

Tissue culture 12-well plates were seeded with 4 × 10^5^ MDA-MB 231 cells and left for 24 h in Dulbecco's Modified Eagle Medium (DMEM) with 10 % fetal bovine serum (FBS). The medium was removed and replaced with 1.5 mL of serum-depleted medium (0 % FBS). Three groups were evaluated:-

A. **Free**^**52**^**Mn control:** 60 μL buffered [^52^Mn]MnCl_2_ made up to 600 μL with serum-depleted medium.
B. **[**^**52**^**Mn]Mn-oxine control**: Solution of [^52^Mn]Mn-oxine (synthesised as previously reported [25]) made up to 600 μL with serum-depleted medium.
C. ^**52**^**Mn-(1) &**^**52**^**Mn-(2):** The appropriate [^52^Mn]Mn-porphyrin in 60 μL DMSO (synthesised using the microwave radiosynthetic method) was made up to 600 μL with serum-depleted medium.

Aliquots (approximately 10 kBq, 100 μL) from each group were added to each well (in triplicate/duplicate per group) which were then incubated at 37 °C for 30 min. Subsequently, the cell medium was removed and the cells washed with PBS (1 mL) and trypsin (150 μL) was added and the cells incubated for 2 min to allow trypsinisation. Cell medium (350 μL) was then added and the cells re-suspended. The counts for the resuspended cells and the cell supernatant plus PBS washes were measured and the labelling efficiency calculated.

### 5.6 Cellular retention of [^52^Mn]Mn-porphyrins and [^52^Mn]Mn-oxine in MDA MB-231 cells

The cells labelled with groups B and C were re-plated on a 6-well plate and cell medium (2 mL) was added. After incubation at 37 °C for 24 h, the cell medium was removed, and the cells washed with PBS (2 mL) and trypsin (300 μL) was added and the cells incubated for 2 min to allow trypsinisation. Cell medium (700 μL) was then added and the cells resuspended. The counts for the resuspended cells and the cell supernatant plus PBS washes were measured in a gamma counter.

### 5.7 Cell viability of MDA MB-231 cells labelling with [^52^Mn]Mn-porphyrins

Cells were seeded as described above. Two groups were evaluated for viability:-

A. **[**^**52**^**Mn]Mn-porphyrins**: ^52^Mn-(**1**) or ^52^Mn-(**2**) in 60 μL DMSO (synthesised using the microwave radiosynthetic method) and made up to 600 μL with serum-depleted medium.
B. **Unlabelled control**: 60 μL DMSO made up to 600 μL with serum-depleted medium.

**For cell viability post-labelling:** aliquots (100 μL) from each group were added to each well. These were then incubated at 37 °C for 30 min. After the labelling of the MDA-MB-231 cells, the cell medium was removed, and the cells washed with PBS (1 mL). The cell medium and PBS washes were combined in a 15 mL falcon and trypsin (150 μL) was added and the cells incubated for 2 min to allow trypsinisation. Cell medium (350 μL) was then added to the wells and the cells re-suspended and combined with cell medium and PBS washes, followed by centrifugation at 200*g* for 5 min. The supernatant was removed, and the cell pellet resuspended in cell medium (1 mL). An aliquot (10 μL) was taken and mixed with trypan blue solution (10 μL). The number of dead and alive cells was then counted on a haemocytometer and the % cell viability calculated from the proportion of the two.

**For cell viability at 24 h post-labelling:** the procedure described above was carried out for groups A & B and the cells were re-plated on a 6-well plate and cell medium (2 mL) was added and incubated for 24 h at 37 °C. The above procedure was then carried out but with differing amounts of PBS (2 mL), trypsin (300 μL) and cell medium (700 μL).

## Declarations and acknowledgements

The work of P. J. G, K. M. N, G. P. K and R. T. M. R was supported by the EPSRC programme for next generation molecular imaging and therapy with radionuclides (EP/S032789/1), the Wellcome EPSRC Centre for Medical Engineering at KCL [grant number WT 203148/Z/16/Z], a CRUK Multidisciplinary Project Award [grant number C48390/A21153], the King's College London & Imperial College London EPSRC Centre for Doctoral Training in Medical Imaging [EP/L015226/1], the KCL/UCL Comprehensive Cancer Imaging Centre funded by CRUK and EPSRC in association with the MRC and DoH (England), the Medical Research Council Confidence in Concepts scheme, the Experimental Cancer Medicine Centre at KCL, the KHP/KCL CRUK Cancer Centre, a Wellcome Trust Multiuser Equipment Grant: A multiuser radioanalytical facility for molecular imaging and radionuclide therapy research [212885/Z/18/Z], the National Institute for Health Research (NIHR) Biomedical Research Centre based at Guy's and St Thomas' NHS Foundation Trust and KCL [grant number IS-BRC-1215-20006], the MRC Doctoral Training Programme, the Research England Confidence in Collaboration scheme. S. M. A. P and M. M. P thank the funding of the FCT - 10.13039/501100001871Foundation for Science and Technology, I.P., under projects UIDB/00313/2020 and PTDC/QUI-OUT/ 27996/2017 (DUALPI). The views expressed are those of the authors and not necessarily those of the NHS, the NIHR or the Department of Health.

## Appendix A Supplementary data

Supplementary material Image 1
