# Supplementary material for: Microwave-assisted synthesis of [52Mn]Mn-porphyrins: Applications in cell and liposome radiolabelling
Source: Nucl Med Biol. 2022 Nov-Dec;114-115:6–17. doi: 10.1016/j.nucmedbio.2022.08.006 (PMC10236072; doi:10.1016/j.nucmedbio.2022.08.006)
Supplement: Supplementary file 1 — Supplementary material [file mmc1.docx]

***Electronic Supplementary Information***

**Microwave-assisted synthesis of [^52^Mn]Mn-porphyrins:** **applications in cell and liposome radiolabelling**

Peter J. Gawne^a1^; Sara M. A. Pinto^b^, Karin M. Nielsen^a^, George P. Keeling^a^ Mariette M. Pereira^b^, and Rafael T. M. de Rosales^a^*.

^a^ School of Biomedical Engineering and Imaging Sciences, King’s College London, St Thomas’ Hospital, London, UK.

^b^ Department of Chemistry, University of Coimbra, 3004-535 Coimbra, Portugal

*Corresponding Author (rafael.torres@kcl.ac.uk)

^1^ Present Address: Laboratory of Nanotechnology for Precision Medicine, Fondazione Istituto Italiano di Tecnologia, Genoa, Italy.

### Supplementary Methods

### Synthesis of the non-radioactive Mn-porphyrins

*Synthesis of porphyrin* ***1***

A mixture of 5,10,15,20-(pentafluorophenyl)porphyrin (140 mg, 0.14 mmol) and PEG500 (mono-methyl polyethyleneglycol – Mw = ∼500 Da) (65 mg, 0.1293 mmol) was dissolved in 5 mL of dry DMF. After the addition of NaH (21 mg, 0.862 mmol) the reaction mixture was left at 80ºC for 48 h. The reaction mixture was extracted several times with dichloromethane/water and dichloromethane/aqueous 1 M K_2_CO_3_ and the organic layer was dried over anhydrous sodium sulfate and concentrated under vacuum. The porphyrin crude was purified via aluminium oxide column chromatography using first CH_2_Cl_2_ followed by CH_2_Cl_2_/5% MeOH. After evaporation, pure porphyrin **1** was obtained in 48.9 % yield. NMR ^1^H (400 MHz, CDCl_3_) δ, ppm: 8.91 (m, 8H); 3.63-3.30 (m, 44 H_PEG_); -2.90 (s, 2 H_NH_). ^19^F NMR (376 MHz, CDCl_3_) δ, ppm: -136.46 to -136.53 (dd, 6F); -140.10 to -140.16 (m, 3F); -151.21 to -152.07 (m, 4F); -161.31 to -161.54 (m, 6F). MS (ESI-TOF): [M]^+^  calc. m/z: 1471.3714; found: 1472.3791 (polydisperse distribution).

#### Synthesis of Mn-(**1**)

Porphyrin **1** (10 mg, 6.8 µmol, 1 equiv.) was dissolved in acetic acid (2 mL) and sodium acetate (55.7 mg, 0.68 mmol, 100 equiv.) in acetic acid (1 mL) was added. Mn(OAc)_2_.4H_2_O (11 mg, 47.6 µmol, 7 equiv.) in acetic acid (1 mL) was added and the solution heated at 80 ^o^C for 2.5 h. The solution was then cooled and taken up in CHCl­_3_ (20 mL) and washed with H_2_O (3 x 20 mL). The organic layer was then dried with MgSO_4_, filtered and the solvent removed *in vacuo* to leave a yellow-brown solid (9.2 mg, 85.3% yield). MS (ESI): m/z [M-C_17_H_37_O_8_]^+^  calc.: 1156.0556; found: 1156.1996 (fragmentation of PEG chain)

#### Synthesis of Mn-(**2**)

Porphyrin **2** (10 mg, 8.9 µmol, 1 equiv) was dissolved in DMF (8 mL), Mn(OAc)_2_.4H_2_O (15.2 mg,  62.3 µmol, 7 equiv.) in DMF (1 mL) was added and the mixture was heated at 120 ^o^C for 24 h. The solution was then cooled and taken up in DCM (20 mL) and washed with H_2_O (3 x 30 mL). The organic layer was then dried with MgSO_4_, filtered and the solvent removed *in vacuo* to leave a brownish solid (8.6 mg, 84.2 % yield). MS (ESI): [M]^+^ calc. m/z: 1183.0473; found: 1183.0464;

#### Synthesis of Mn-(**3**)

Porphyrin **3** (10 mg, 7.3 µmol, 1 equiv) was dissolved in DMF (8 mL), Mn(OAc)_2_.4H_2_O (12.5 mg, 51.1 µmol, 7 equiv.) in DMF (1 mL) was added and the mixture was heated at 120 ^o^C for 24 h. An additional amount of Mn(OAc)_2_.4H_2_O (23.3 mg, 94.9 µmol, 13 equiv.) in DMF (2 mL) was added and the mixture was heated at 120 ^o^C for a further 6 d. The solution was then cooled and taken up in DCM (20 mL) and washed with H_2_O (3 x 30 mL). The organic layer was then dried with MgSO_4_, filtered and the solvent removed *in vacuo* to leave a red solid (8.8 mg, 81.2 % yield). MS (ESI): [M]^+^ m/z: 1428.9; found: 1428.3

#### Synthesis of Mn-(**5**)

Porphyrin **5** (10 mg, 10.7 µmol, 1 equiv) was dissolved in DMF (8 mL), Mn(OAc)_2_.4H_2_O (15.7 mg, 74.9 µmol, 7 equiv.) in DMF (1 mL) was added and the mixture was heated at 120 ^o^C for 24 h. The solvent was then removed *in vacuo* to leave a black solid. MS (ESI): [M]^2-^ calc. m/z: 492.9; found: 492.3; [M]^3-^ calc. m/z: 328.3; found: 327.9

#### Synthesis of Mn-(**6**)

Porphyrin **6** (10 mg, 9.27 µmol, 1 equiv) was dissolved in DMF (8 mL), Mn(OAc)_2_.4H_2_O (13.6, 64.9 µmol, 7 equiv.) in DMF (1 mL) was added and the mixture was heated at 120 ^o^C for 24 h. The solvent was then removed *in vacuo* to leave a red-pink solid. MS (ESI): [M]^3-^ calc. m/z: 376.6 found: 375.9

### Characterisation of the non-radioactive Mn-porphyrins

#### UV-vis spectrometry of the non-radioactive Mn-porphyrins

An aliquot (4 – 10 µL) of the reaction mixture was taken and made up to 0.8 mL with DMF (for porphyrins **3, 5** & **6**) or H_2_O (for porphyrin **1**) in a UV cuvette (0.8 mL, 1 cm pathlength, plastic). The UV-vis spectrum was then taken at room temp., subtracting a blank spectrum of DMF (for porphyrins **3, 5** & **6**) or H_2_O (for porphyrin **1**). Absorbance was then normalised with the y value for 700 nm being set to 0.

#### HPLC characterisation of the non-radioactive Mn-porphyrins and free base porphyrins

Column used: Eclipse XDB-C18 (5 µm; 4.6 x 150 mm). Flow rate: 1 mL/min

**Solvent system 1: A** = H_2_O + 0.1 % TFA; **B** = Acetonitrile + 0.1 % TFA.

| Time (mins) | **A%** | **B%** |
| --- | --- | --- |
| 0 | 95 | 5 |
| 5 | 95 | 5 |
| 20 | 10 | 90 |
| 25 | 10 | 90 |
| 26 | 95 | 5 |
| 30 | 95 | 5 |

**Solvent system 2: A** = H_2_O + 0.1 % TFA; **B** = Acetonitrile + 0.1 % TFA.

| Time (mins) | **A%** | **B%** |
| --- | --- | --- |
| 0 | 95 | 5 |
| 2 | 95 | 5 |
| 20 | 5 | 95 |
| 25 | 5 | 95 |
| 26 | 95 | 5 |
| 30 | 95 | 5 |

### Serum association of free ^52^Mn and ^52^Mn-(1)

An aliquot of the purified ^52^Mn-(**1**) prepared was diluted with H_2_O to give a 10% EtOH solution and filtered with a 0.2 µm filter. An aliquot was then diluted 1:2 with human serum (0.2 µm filtered) and incubated at 37 ^o^C for 1 min, 10 min, 30 min, 16 h and 24 h. At each timepoint, a 20 µL aliquot was taken and EtOH (180 µL) to precipitate the serum proteins. The mixture was then centrifuged at 5000 rpm for 2 mins and the supernatant and precipitated proteins separated for γ-counting.

For the free ^52^Mn control: An aliquot of ^52^MnCl_2_ buffered with 0.5 M NH_4_OAc was diluted 1:2 with human serum (0.2 µm filtered) and incubated at 37 ^o^C for 1 min, 10 min, 30 min and 24 h. At each timepoint, a 20 µL aliquot was taken and EtOH (180 µL) to precipitate the serum. The mixture was then centrifuged at 5000 rpm for 2 mins and the supernatant and precipitated proteins separated for γ-counting.

**Table S1**: Table summarising the various radiolabelling conditions tested for the synthesis of ^52^Mn-(2). ^a^ this temperature was achieved using the microwave synthesiser unit.

| Porphyrin conc. (µM) | Reaction time (h) | Reaction Temperature (^o^C) | Radiochemical Yield (%) |
| --- | --- | --- | --- |
| 200 | 6 | 20 | 0 |
| 200 | 2 | 65 - 80 | 0 |
| 200 | 48 | 70 | 15.6 ± 2.3 % |
| 500 | 48 | 70 | 52.8 ± 3.2 % |
| 500 | 1 | 165^a^ | 79.7 ± 1.8 %. |
| 600 | 1 | 165^a^ | 97.3 ± 0.9 % |

**Table of Supplementary Figures**

Figure S1: UV-vis spectroscopy of the porphyrin ligands and their Mn(III) complexes. 8

Figure S2: HPLC traces for the porphyrin ligands and their non-radioactive Mn complexes. 9

Figure S3: Stability of ^52^Mn-(1) and ^52^Mn-(2) radiocomplexes in cell medium. 10

Figure S4: Radio-HPLC of the ^52^Mn-(5) and its corresponding non-radioactive Mn-porphyrin 11

Figure S5: ^1^H NMR of MonoPEG porphyrin 1. 12

Figure S6: ^19^F NMR (b) of MonoPEG porphyrin 1. 13

Figure S7: Mass spectra (ESI-TOF) of MonoPEG porphyrin 1 16

Figure S8: Mass spectra (ESI-TOF) of Mn-(1) 17

Figure S9: Mass spectra (ESI-TOF) of Mn-(2) 18

Figure S10: Mass spectra (LC-MS ESI+) for Mn-(3) 20

Figure S11: Mass spectra (LC-MS ESI-) for Mn-(5) 20

Figure S12: Mass spectra (LC-MS ESI-) for Mn-(6) 21


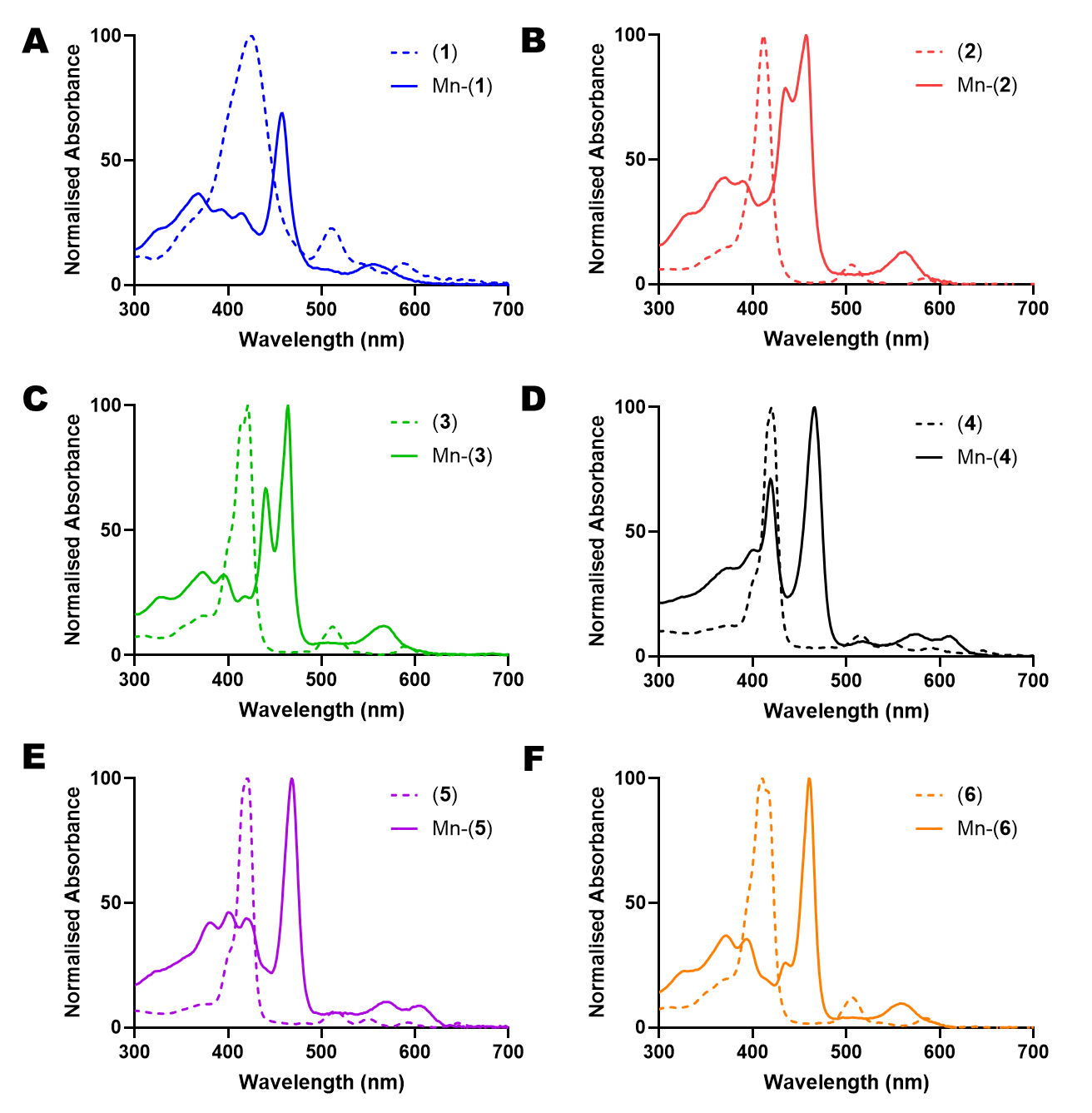


Figure S1: UV-vis spectroscopy of the porphyrin ligands and their Mn(III) complexes.

UV-vis spectrums of the porphyrin ligands (dotted lines) and their corresponding Mn(III) complexes (solid lines). Absorbance is normalised with the Y value at 700 nm being set to 0.


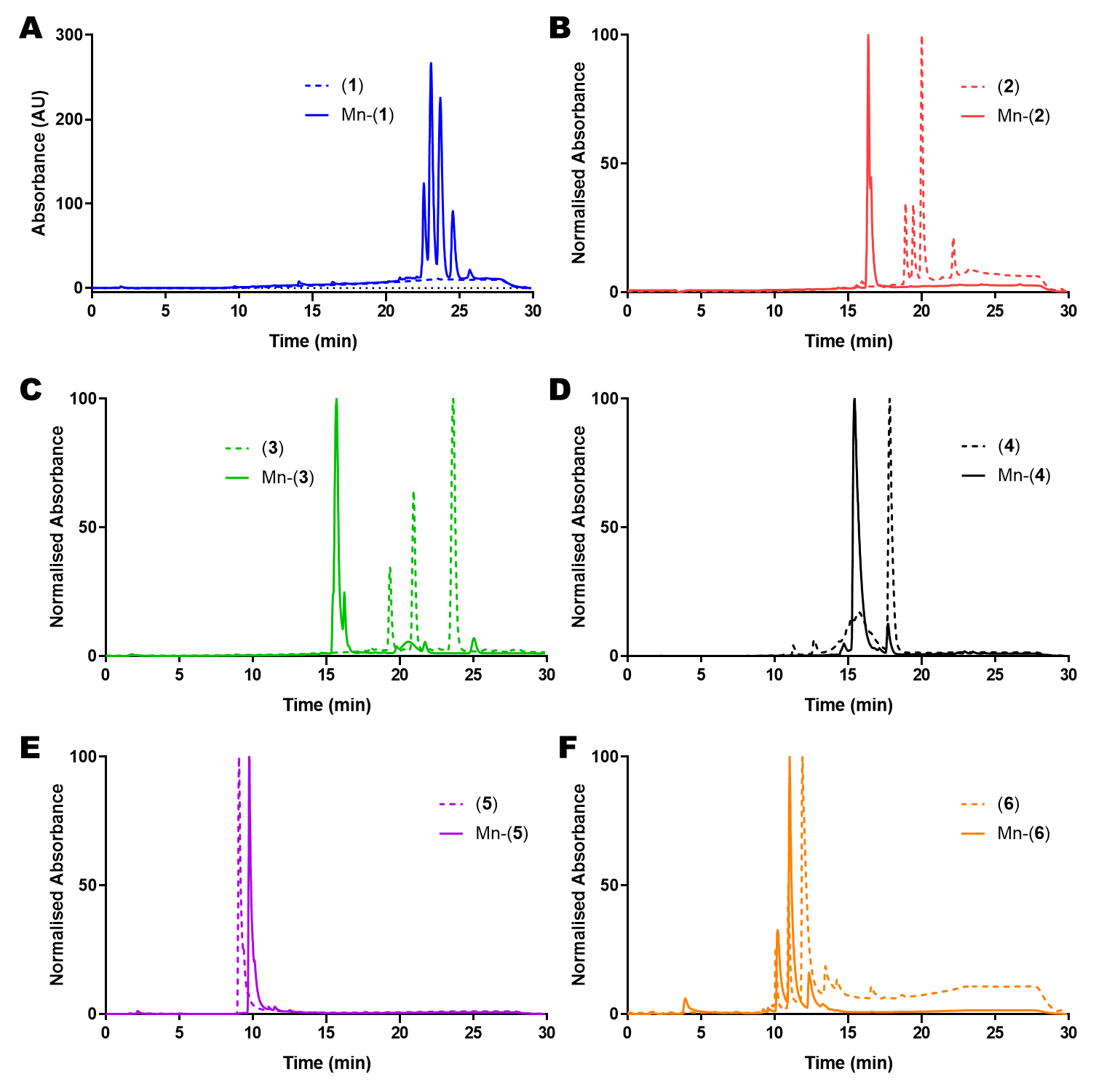


Figure S2: HPLC traces for the porphyrin ligands and their non-radioactive Mn complexes.

HPLC traces for **A)** porphyrin **1** and Mn-(**1**); **B)** porphyrin **2** and Mn-(**2**); **C)** porphyrin **3** and Mn-(**3**); **D)** porphyrin **4** and Mn-(**4**); **E)** porphyrin **5** and Mn-(**5**) and **F)** porphyrin **6** and Mn-(**6**). Note that the HPLC gradient used for porphyrin **3** and its complex is distinct from the others. Additionally, the absence of a signal for porphyrin **1** is likely due to the high lipophilicity of the ligand; resulting in the compound not eluting in the HPLC gradient used. The less lipophilic Mn-(**1**) can clearly be observed.


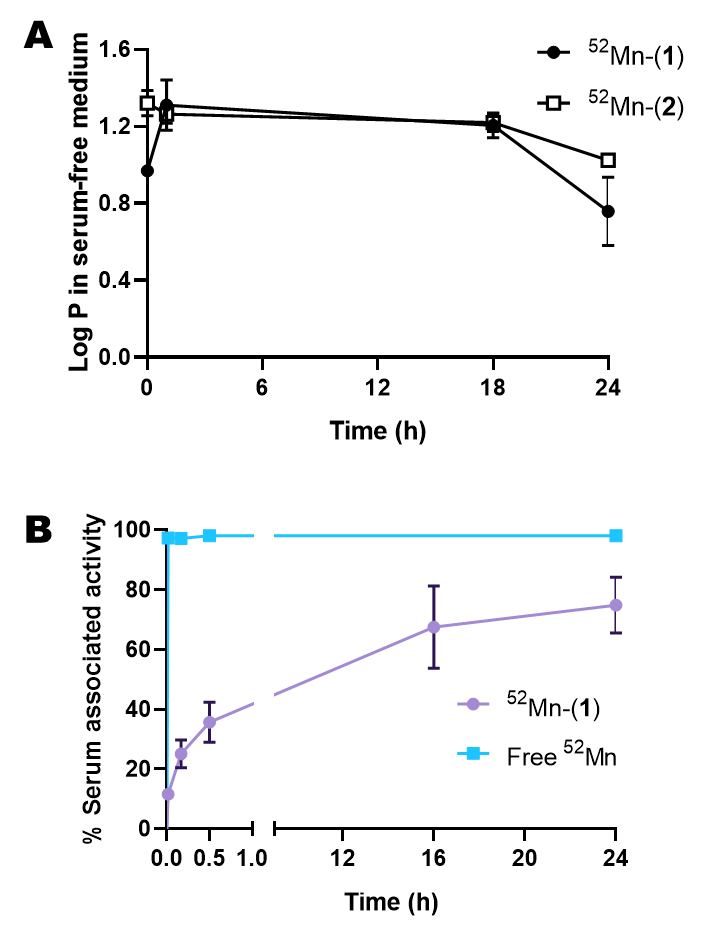


Figure S3: Stability of ^52^Mn-(1) and ^52^Mn-(2) radiocomplexes in cell medium.

**A)** Human serum associated radioactivity over time of ^52^Mn-(**1**) and free ^52^Mn (n = 3) **B)** Stability of ^52^Mn-(**1**) and ^52^Mn-(**2**) radiocomplexes in cell medium (DMEM) at room temp., based on the log P values (n = 2). All error bars represent mean ± SD.


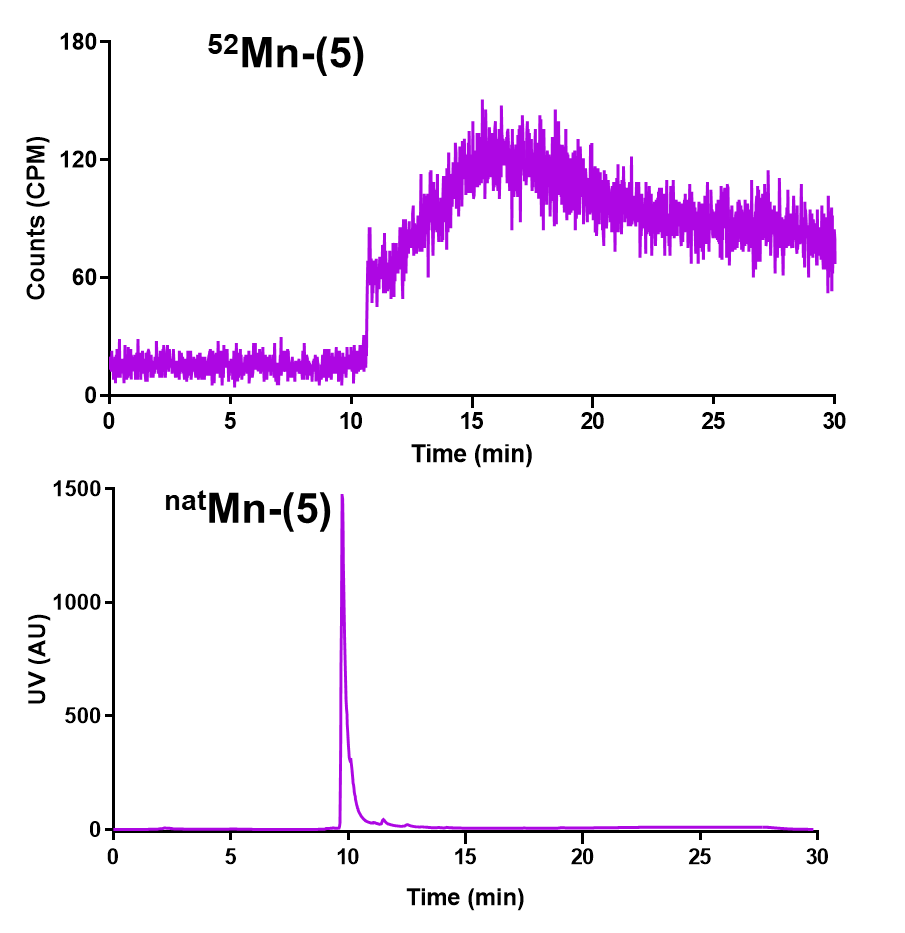


Figure S4: Radio-HPLC of the ^52^Mn-(5) and its corresponding non-radioactive Mn-porphyrin

Figure S5: ^1^H NMR of MonoPEG porphyrin 1.


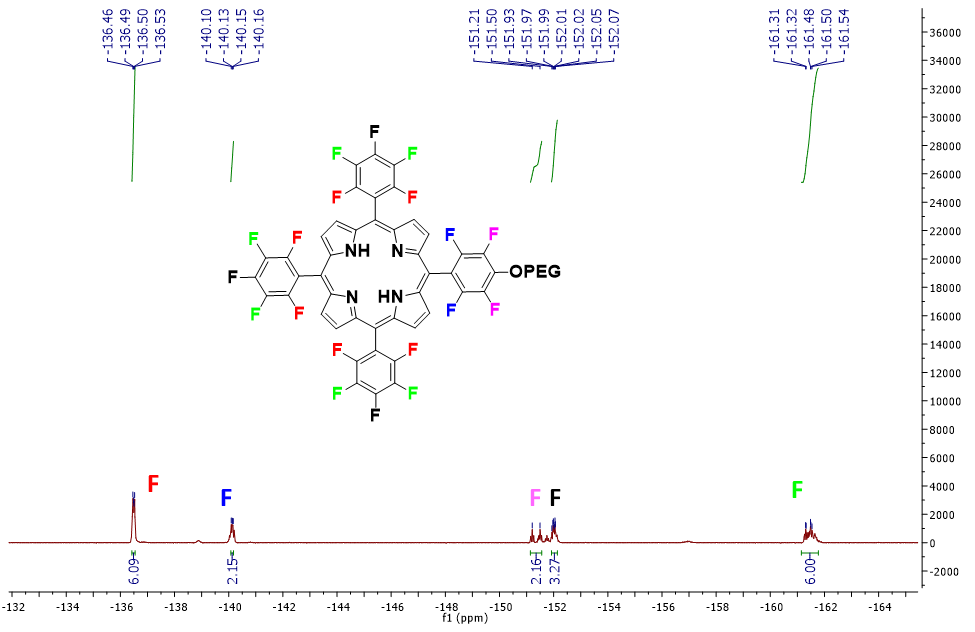


Figure S6: ^19^F NMR (b) of MonoPEG porphyrin 1.


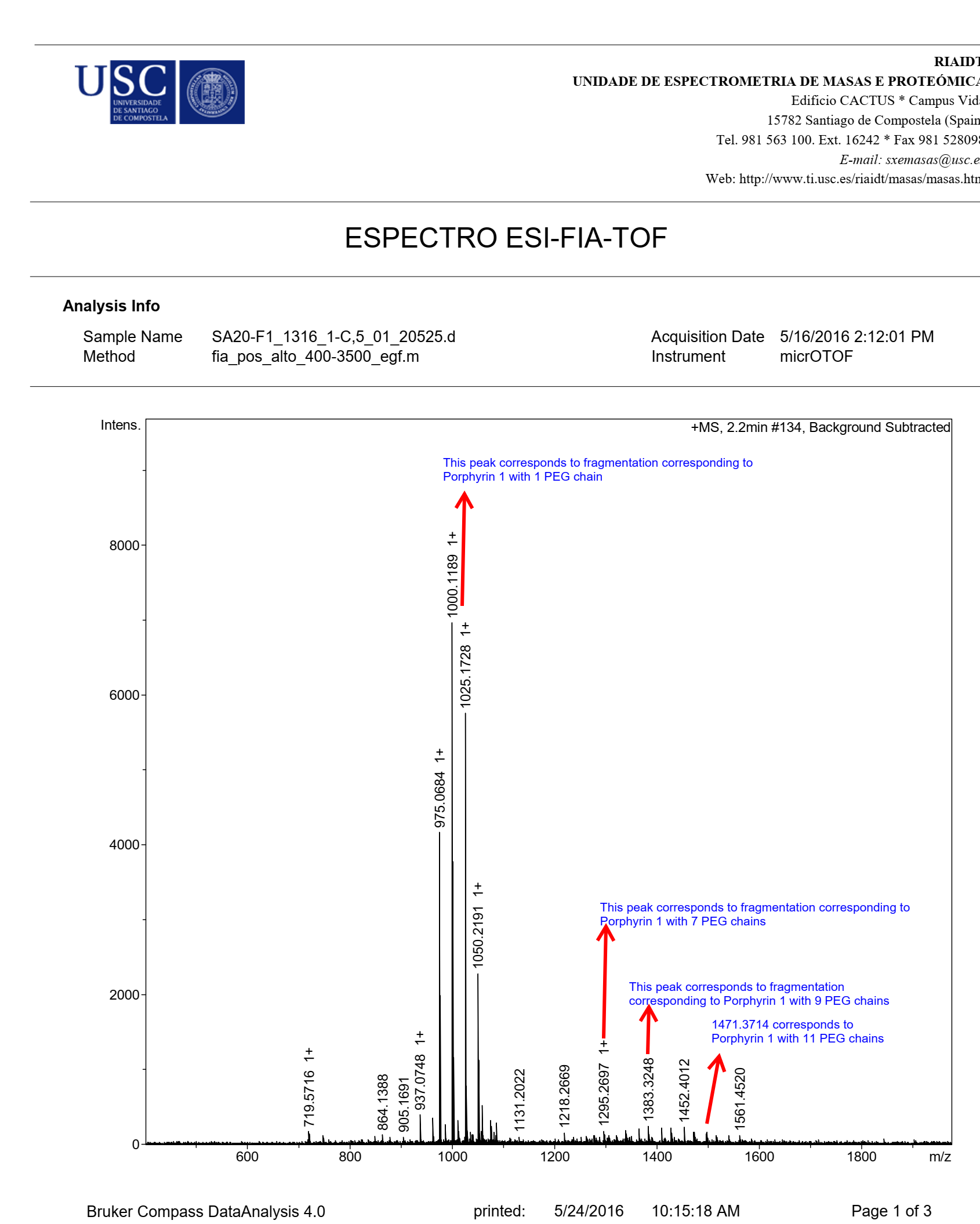

Figure S7: Mass spectra (ESI-TOF) of MonoPEG porphyrin 1


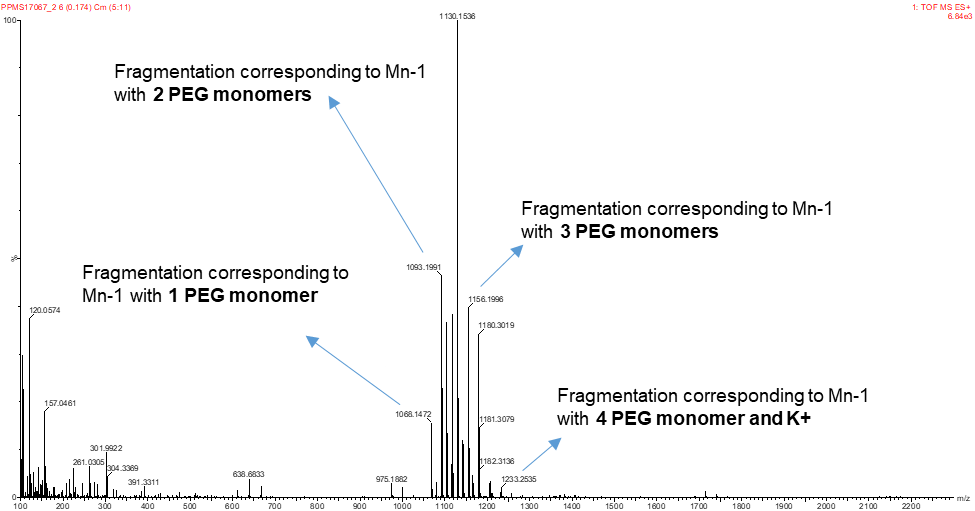


Figure S8: Mass spectra (ESI-TOF) of Mn-(1). Note: Mass spectrometry resulted in fragmentation of the PEG polymer (10/11 monomer chains). The masses relating to Mn-(1) with the corresponding number of PEG monomers are shown.


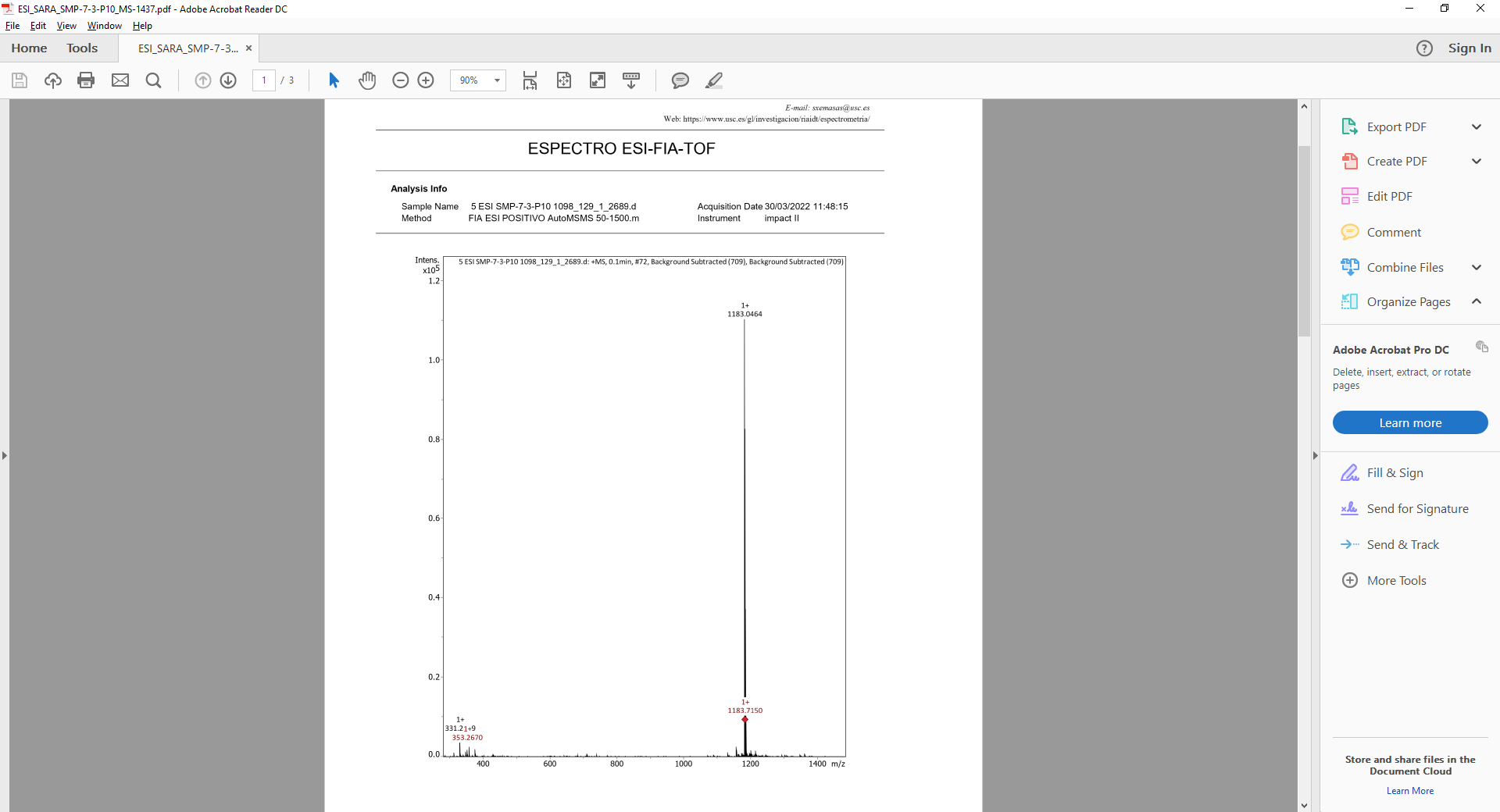


Figure S9: Mass spectra (ESI-TOF) of Mn-(2)


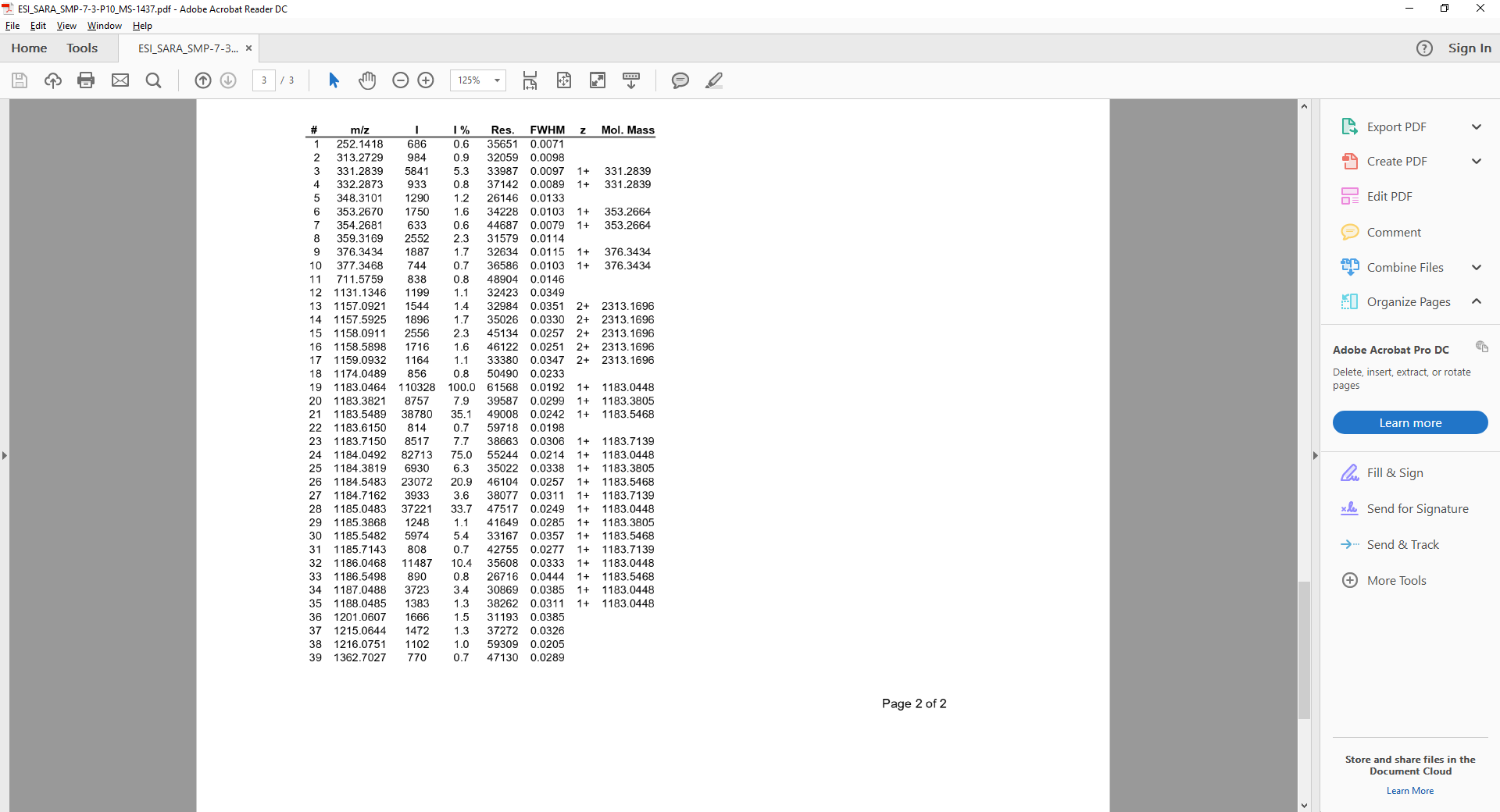


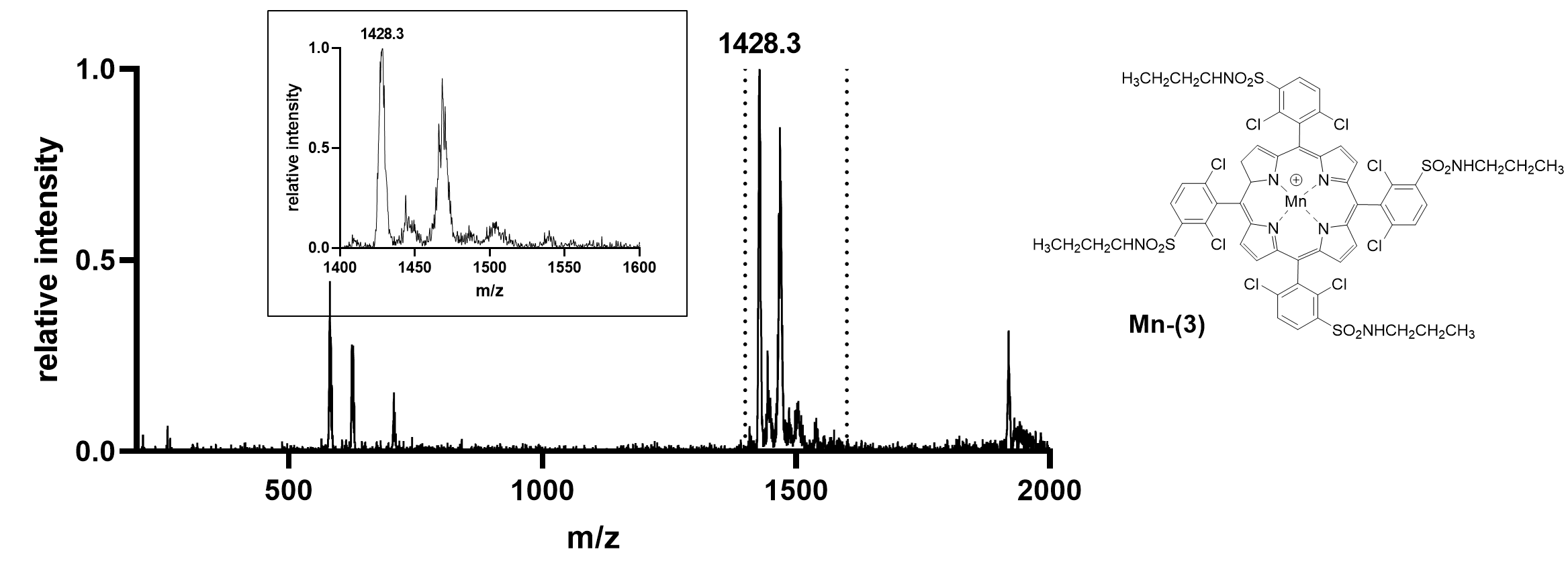


Figure S10: Mass spectra (LC-MS ESI+) for Mn-(3)


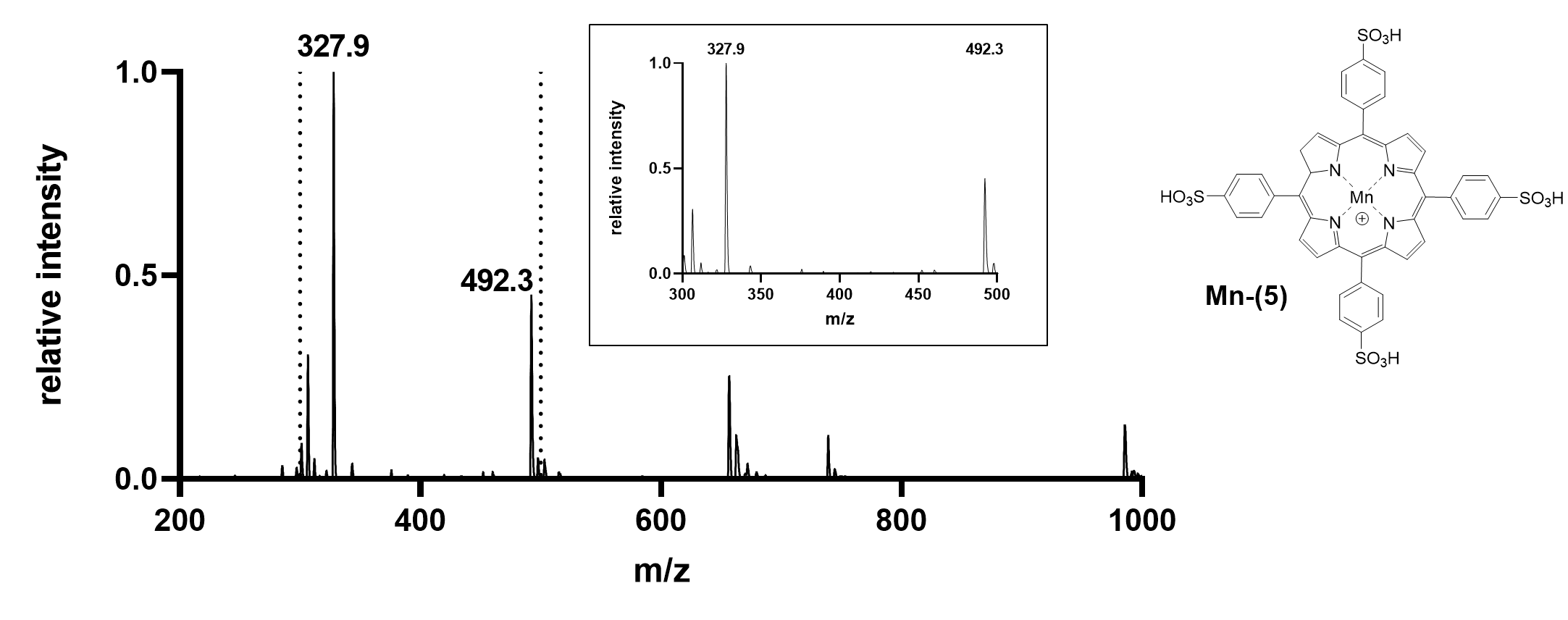


Figure S11: Mass spectra (LC-MS ESI-) for Mn-(5)


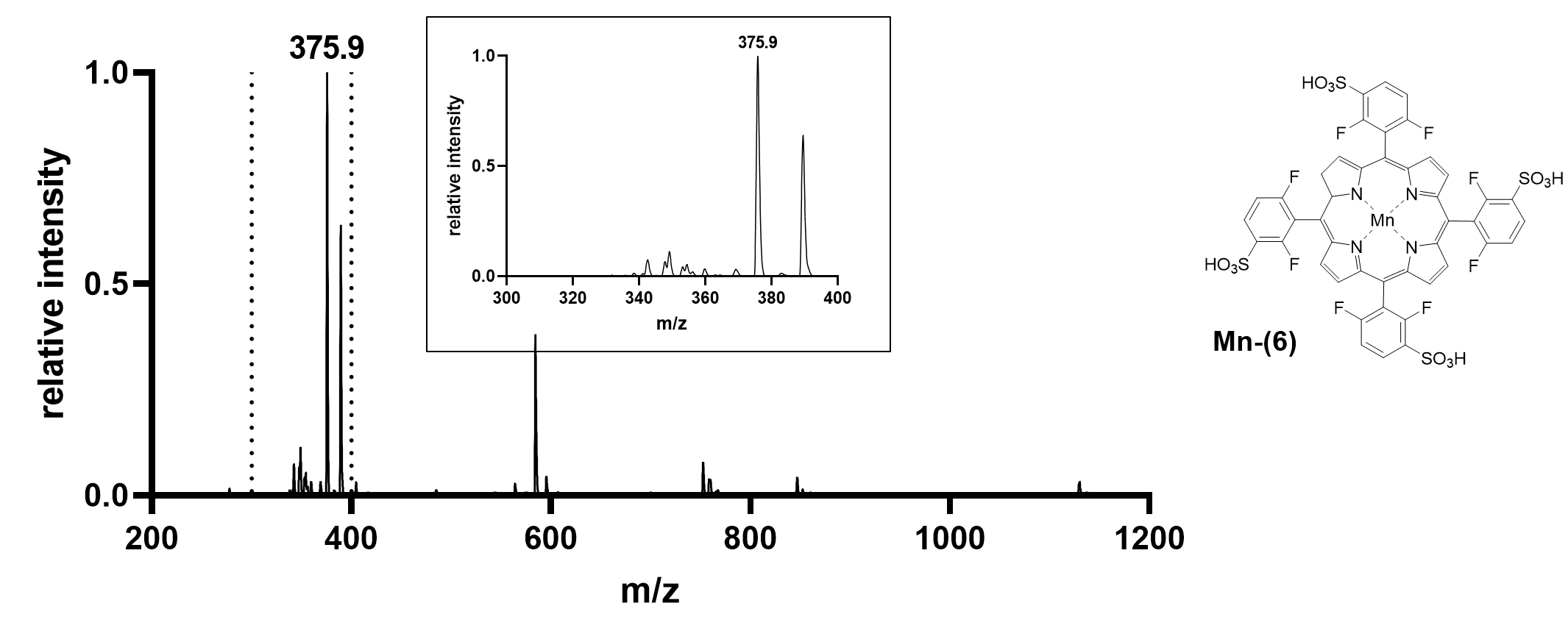


Figure S12: Mass spectra (LC-MS ESI-) for Mn-(6)
